# Defining and Assessing the Syndrome of Moral Injury: Initial Findings of the Moral Injury Outcome Scale Consortium

**DOI:** 10.3389/fpsyt.2022.923928

**Published:** 2022-07-05

**Authors:** Brett T. Litz, Rachel A. Plouffe, Anthony Nazarov, Dominic Murphy, Andrea Phelps, Alanna Coady, Stephanie A. Houle, Lisa Dell, Sheila Frankfurt, Gadi Zerach, Yossi Levi-Belz, The Moral Injury Outcome Scale Consortium, Andrea Ashbaugh

**Affiliations:** ^1^Psychiatry Department, VA Boston Healthcare System, Boston University, Boston, MA, United States; ^2^Psychiatry Department, The MacDonald Franklin Operational Stress Injury Research Centre, University of Western Ontario, London, ON, Canada; ^3^King's Centre for Military Health Research, Kings College London, London, United Kingdom; ^4^Psychology Department, Phoenix Australia - Centre for Posttraumatic Mental Health, University of Melbourne, Melbourne, VIC, Australia; ^5^VA Boston Healthcare System, Boston, MA, United States; ^6^Psychology Department, University of Ottawa, Ottawa, ON, Canada; ^7^Center of Excellence for Research on Returning War Veterans, Central Texas Veterans Health Care System, Temple, TX, United States; ^8^Psychology Department, Ariel University, Ariel, Israel; ^9^Behavioral Sciences Department, Ruppin Academic Center, Emek Hefer, Israel

**Keywords:** moral injury syndrome, moral injury outcome scale, multinational, psychometric evaluation, moral injury outcome scale consortium

## Abstract

Potentially morally injurious events (PMIEs) entail acts of commission (e.g., cruelty, proscribed or prescribed violence) or omission (e.g., high stakes failure to protect others) and bearing witness (e.g., to grave inhumanity, to the gruesome aftermath of violence), or being the victim of others' acts of commission (e.g., high stakes trust violations) or omission (e.g., being the victim of grave individual or systemic failures to protect) that transgress deeply held beliefs and expectations about right and wrong. Although there is a proliferation of interest in moral injury (the outcome associated with exposure to PMIEs), there has been no operational definition of the putative syndrome and no standard assessment scheme or measure, which has hampered research and care in this area. We describe an international effort to define the syndrome of moral injury and develop and validate the Moral Injury Outcome Scale (MIOS) in three stages. To ensure content validity, in Stage I, we conducted interviews with service members, Veterans, and clinicians/Chaplains in each country, inquiring about the lasting impact of PMIEs. Qualitative analysis yielded six operational definitions of *domains of impact* of PMIEs and components within domains that establish the parameters of the moral injury syndrome. From the domain definitions, we derived an initial pool of scale items. Stage II entailed scale refinement using factor analytic methods, cross-national invariance testing, and internal consistency reliability analyses of an initial 34-item MIOS. A 14-item MIOS was invariant and reliable across countries and had two factors: *Shame-Related* (SR) and *Trust-Violation-Related* (TVR) Outcomes. In Stage III, MIOS total and subscale scores had strong convergent validity, and PMIE-endorsers had substantially higher MIOS scores vs. non-endorsers. We discuss and contextualize the results and describe research that is needed to substantiate these inaugural findings to further explore the validity of the MIOS and moral injury, in particular to examine discriminant and incremental validity.

## Introduction

The idea that people can be lastingly psychologically and socially affected by their own or others' transgressive behavior is as old as humanity. It is only recently that these age-old concepts have been considered as clinically relevant social, biological, and psychological problems. The term that is used to describe the outcome of these transgressive harms is moral injury (MI). As is the case with the distinction between stressors and stress, transgressive experiences are best construed as *potentially* morally injurious events (PMIEs), rather than inherently and enduringly impairing. PMIEs entail acts of commission or omission by oneself (e.g., cruelty, failure to prevent serious injury), or bearing witness to, learning about, or being the direct victim of acts of commission or omission of others (e.g., high stakes betrayal by an individual or institution, witnessing cruel behavior), that transgress deeply held moral beliefs and expectations (1–4). MI has been most studied in Veterans for good reason; a sizeable minority (24%−40%) of deployed service members (SMs) and combat Veterans report exposure to PMIEs during their military service (2–4).

Litz et al. (1) posited that PMIEs are potentially harmful because they can undermine foundational beliefs about the goodness and trustworthiness of oneself or others, causing functionally impairing psycho-social-spiritual problems. Currier et al. (5), Farnsworth et al. (6), Jinkerson (7), and Litz et al. (1) posited that there are areas of overlap and distinction between MI and other mental and behavioral health outcomes. We hypothesized that the outcomes associated with exposure to PMIEs closely resemble PTSD, as is the case with the potential aftermath of *any* high magnitude life stressor. When PMIEs are impairing (a person putatively is experiencing MI), memories of the experiences can be haunting (intrusively reexperienced) and individuals are motivated to avoid reminders of the event(s) because they cue painful functionally impairing moral emotions, namely shame, guilt, anger, and disgust (1). Additional overlapping PTSD symptoms entail restricted range of non-moral emotions, disinterest in pleasurable activities, and detachment from others, which are also symptoms of depression. MI is posited to have two non-exclusive forms, namely, internalizing outcomes associated with personal transgressive acts and externalizing outcomes associated with being the victim of other's transgressions (8). The distinctive features of MI are posited to be unique enduring changes in self-schemas and beliefs about others that reflect over-accommodation of moral violation, culpability, or expectations of injustice, as well as estrangement, and risky (e.g., reckless) or self-destructive behaviors (1). Although a sizeable percentage of traumatic events endorsed by SMs and war Veterans with PTSD entail morally injurious events, MI is uniquely associated with additional symptoms and problems among PTSD cases (3). However, a frequent false assumption is that for PMIEs to substantively impact outcomes, these events are de facto Criterion-A traumas or take place within a life-threatening context. While PMIEs can be classified as traumatic events (e.g., sexual assaults in the military), many do not involve life-threat and/or sexual assault (e.g., drone strikes, humiliation of a prisoner of war, high stakes trust violations). Finally, although MI and PTSD overlap as described above, some apparently overlapping symptoms may differ functionally [e.g., vigilance about potential betrayal, detachment or anger as a means to avoid shame; (6)].

Reports of PMIEs have also been shown to be associated with suicidal ideation and behavior, anger/aggression, depression/hopelessness, guilt/self-blame, alcohol misuse (3, 4, 9, 10), impairments in occupational and social/relationship functioning (11), and spiritual or existential conflicts or deficits (12). However, these studies have been hampered by typically small samples of convenience, and the findings are generally of very small magnitude and have questionable replicability. Generally, research about MI and efforts to treat the putative clinical aftermath of exposure to PMIEs are hindered by a lack of consensus about the problems uniquely and reliably associated with exposure to PMIEs (the putative syndrome of MI) and the lack of a gold standard measure of MI as an outcome. The lack of a gold standard measure is particularly problematic with respect to identifying clinical cases of MI, planning treatment for those cases, tracking change in MI symptoms over the course of treatment, and evaluating effectiveness. Although treatments have been developed to purportedly target MI, this work has been somewhat cart before horse. Without a gold standard measure of MI as an outcome, it is impossible to demonstrate efficacy. Finally, advancements in the field have been particularly hampered by the absence of qualitative evaluations of the lived experiences of individuals exposed to moral harms. Instead, most studies that have generated ideas about the parameters of the MI construct have interviewed putative experts and clinicians or administered existing mental and behavioral health questionnaires. Given the lack of consensus about, and rhetorical fuzziness associated with MI (8, 13), expert opinion is widely varying and has uncertain validity. Consequently, the lack of qualitative data on how people suffer after exposure to transgressive acts represents a particularly significant knowledge gap in the field.

There are two extant measures of MI as an outcome, namely the Moral Injury Symptom Scale—Military Version [MISS-M (14)] and the Expressions of Moral Injury Scale—Military Version [EMIS-M (5)]. The MISS-M was created by compiling items from existing outcome scales that the authors judged to be face valid. Additional items that putatively assessed domains not assessed in existing scales were derived by the authors or from other studies. The initial scale was subjected to exploratory and confirmatory factor analyses in a sample of Veterans and active-duty SMs. The authors failed to follow state-of-the-art steps in test construction and validation (15) and failed to establish content validity (16), to ensure the meaningfulness of scale content.

The items for the EMIS-M (5) were developed in a four-stage process that included: (a) a literature review and consultation with three putative subject matter experts to identify MI; (b) an unspecified review of existing measures of relevant constructs; (c) creating an initial pool of items and soliciting feedback from clinicians and researchers; and (d) refining the item pool in consultation with putative subject matter experts. The initial scale was subjected to exploratory factor analysis in a college student Veteran sample. The EMIS-M correlated positively with PTSD and depression symptoms and was inversely associated with social support, hope, and gratitude in the student Veteran sample. The authors generated content from existing scales and by appealing to putative experts. We argue that this constrains content validity, which should entail consulting the target population to ensure the meaningfulness and comprehensiveness of scale content (16). Another problem with the EMIS-M is that the scale items ask respondents to rate symptoms relative to “the military or the military experience,” failing to index symptoms to a specific worst and currently distressing PMIE, which also limits the scale's applicability outside the military context. Without event linkage, the EMIS-M is questionably helpful to clinicians who may wish to target the meaning and implication of a specific event and to track change in MI yoked to the most currently distressing and targeted event. An additional problem with the EMIS-M is that respondents are not asked to rate symptoms within a specific time period. This means that the scale might be assessing traits rather than states and would have difficulty tracking clinical change. Finally, the EMIS-M does not measure the functional impact of the symptoms endorsed, which Litz and Kerig (8) argued is a way of distinguishing moral frustration and distress (resulting from moral challenges and stressors, respectively) from MI (based on exposure to PMIEs).

We describe an international effort to develop and validate a questionnaire measure of MI as an outcome, the Moral Injury Outcome Scale (MIOS). This research was conducted by a consortium comprised of researchers and clinicians working with active-duty military SMs and Veterans in the US, the United Kingdom (UK), Israel, Australia, and Canada. We paid considerable attention to ensuring a high degree of content validity for the MIOS. We conducted qualitative interviews of SMs, Veterans, clinicians (psychologists, social workers, mental health nurses), and Chaplains from each participating country. We then used the results, as well as theory, to generate operational definitions of the cross-country domains of impact of PMIEs (and components within domains) that do not overlap with PTSD or depression, with the dual aim of defining the syndrome of MI and to generate scale items (17). The construct and measure development process were divided into three stages, following the methods described by Haynes et al. (16) and Vogt et al. (18). Stage I included content generation and creation of the initial measure. Stage II entailed scale refinement and invariance testing (e.g., item reduction and revisions of the structure and format of scale). Stage III entailed an additional test of construct validity of the final iteration of the MIOS *via* an examination of convergent validity. (A study of discriminant and incremental validity is in the planning stages).

## Stage I: Item Generation and Initial Scale Construction

We used theory and consensus among consortium members to generate an initial set of domains of impact from exposure to PMIEs. These hypothesized domains of impact were used to generate prompt questions in a semi-structured interview, which was used to substantiate these domains and discover new domains and specific components (elements) within all domains. The aim was to use thematic analysis to capture the phenomenology of participants' lived experience (19), to generate operational definitions of an invariant, best-fitting set of domains of impact and components within domains, from which to generate content-valid items for the MIOS. The domains and components within domains were conceptualized as higher and lower order parts, respectively, of the nomological network that defines the MI construct. A large pool of items was generated from each component definition within each domain, and these were pared down by categorizing whether items well-fit each domain. Then, a card sort task was used to generate an initial item set for the MIOS (some items were re-worded and additional items were generated to fill gaps).

### Methods

#### Procedure and Results

We conducted qualitative semi-structured interviews of SMs, Veterans, and clinicians (and clergy) with experience treating SMs and Veterans across consortium countries. Prior to SM and Veteran interviews, we asked respondents a series of demographic and military history questions, and we asked them to identify and describe the worst and most currently distressing military experience that went against their beliefs about right and wrong.

We generated a semi-structured interview in which we asked SMs and Veterans to describe the ways that their life changed as a result of the worst and most currently distressing PMIE (and we asked clinicians what they observed). The initial prompt questions asked SMs and Veterans to reflect on an initial hypothesized set of domains of impact, namely: (1) *the presence of moral emotions* (e.g., how do you feel now as you are thinking about this event?); (2) *alterations in self-perception* (e.g., how has this event changed the way you see or feel about yourself; the way you care for yourself; your plans for the future?); (3) *social impacts* (e.g., how has this event changed your relationships with family, friends, romantic partners, and co-workers; what about your trust in other people; dealing with authority figures; how close or distant you feel toward others; how you care for others?); and (4) *beliefs about life's meaning and purpose* (e.g., how has this event affected the way you make sense of life and your spirituality or religious beliefs [faith], your understanding of right and wrong, the principles that guide your life?). The interview for clinicians/clergy framed these questions as observations across patients.

Seven SMs, 65 Veterans, 64 clinicians, and 12 Chaplains were interviewed in total (see Supplementary Material for the consortium site contributions). Interviews were audio-taped and transcribed. All efforts were approved by the internal review (ethics) board of the respective sites. The initial qualitative analysis was conducted by two teams, one in the US and one in Canada. In Boston, two trained and well-versed research assistants, and in Ottawa, three clinicians, repeatedly read the transcripts, generated initial codes, and searched for and reviewed themes (20). This process was carried out using NVivo 12 qualitative coding software. The meta-frame for this process was the assumption that PMIEs can adversely affect behavior and ways of thinking, feeling, and relating, and that MI subsumes two separable sub-constructs, namely, the unique aftermath of PMIEs that entail personal actions (or omissions), and the unique aftermath of PMIEs that entail bearing witness to or being directly impacted by the transgressions of others. In addition, raters understood that in theory, self-transgressions were associated with shame, a self-conscious and self-condemning emotion, and others' norm violations produce anger, an other-condemning emotion (1). The two teams met in person to discuss their findings and to generate consensus definitions of themes. We relied on theory and data to identify themes pertinent to generating operational definitions of domains of impact.

The US team coded eight interviews for the initial codebook, one active-duty US SM, four Veterans (one from Australia, two from the UK, and two from the US), and three clinicians (one from Australia, one from the UK, and one from the US). The codebook was then tested at each of the sites on at least four of their local interviews. A priori, consortium members decided that the MIOS should be designed to maximize incremental validity relative to related constructs, such as PTSD and depression. Consequently, at first, coders coded everything that was present in the data and then removed all codes that entailed prototypic DSM-5 diagnostic criteria for PTSD and depression (e.g., intrusive experiences, anhedonia). They also removed codes that described PMIEs and codes suggesting positive outcomes of exposure to PMIEs. An initial codebook was sent to consortium sites so they could code their site-specific data. Consortium members met monthly *via* conference calls to identify gaps and inconsistencies and to further refine the codebook. The final codebook consisted of “themes,” subsequently renamed domains, and “codes,” renamed as components. The domain definitions are presented in Table 1 (components within domains are in Supplementary Material).

**Table 1:** Domains of impact and their operational definitions.

**Domains of impact**					
**Self-perception**	**Moral thinking**	**Social impacts**	**Self-harming/self-sabotaging**	**Impairing moral emotions**	**Beliefs about meaning and purpose**
**Operational definitions of domains**					
Alterations and disruptions in identity, which entail how individuals regard, understand, define, or see themselves (who they are), with respect to their core moral beliefs and what they are capable of	Changes in moral thinking, which entail the person judging situations or others moralistically and with condemnation	Alterations in degree of comfort with others, connectedness, social acceptance/belonging, changes in social behavior (e.g., the frequency and quality of engaging with others), trust in others and expectations of social safety	Deliberate and non-deliberate behaviors that negatively impact functioning, and impair health, personal safety, and quality of life / overall wellbeing	Predominant emotions and moods that arise when thinking of the event or that have been more prevalent since the event, as well as avoidance of emotions. Emotions/moods also include emotional behaviors and physiological reactions	Alterations in individuals' beliefs about life meaning or purpose, which may include religious or spiritual beliefs

Next, separately, each consortium site member generated a large initial pool of scale items that tapped content consistent with the definitions of each component within domains. The non-overlapping items were culled and edited for clarity and simplicity of language, resulting in a set of approximately 300 items. The individuals in each site that had content knowledge or clinical experience pertaining to MI then rated each item from the 300-item pool with respect to whether the item was a “Core” or “Not Core” fit with the operational definition of the respective domains. The 49 items retained were primarily “I statements” to assess personal constructions about behavioral repertoires, ways of thinking (beliefs), ways of feeling, and ways of relating to others that were altered by exposure to PMIEs.

We then created an online card sorting task that included the operational definitions of each domain at the top of the page. A separate group of 19 support staff and research assistants across the consortium, unfamiliar with the MIOS project or MI, were asked to move each item into virtual domain bins. Twenty-seven items in which at least 50% of the raters agreed were retained (six of these were reworded to enhance clarity). The total interrater agreement for these 27 items was moderate [Kappa = 0.45, 95% CI, (0.17, 0.72)]. To ensure that the MIOS covered content that consortium members deemed important, upon reflection, an additional seven items were created (e.g., we determined that the existing content did not capture *the loss of* previously valued aspects of the self or constructions of others; an example item was “I have lost pride in myself”). The original list of items retained from the “core” “not core” process, the 27 items retained from the card sort (as well as the rewording), and the additional seven items are presented in Supplementary Table 1.

Finally, we generated a working instructional set and response framework for the initial 34-item MIOS to be tested in Stage II, incorporating feedback about item content and the form of the MIOS from subject matter and questionnaire design experts, respectively. The first page of the MIOS establishes whether a respondent experienced a PMIE, and if so, respondents are asked to categorize their worst and most currently distressing PMIE (respondents are asked to select “Yes” or “No” in response to the following questions: Did the event involve something you did or failed to do?; Did the event involve observing someone else acting [or failing to act]?; Did the event involve being directly impacted by someone else [or people] acting [or failing to act]?). We then asked respondents to report the year that the event happened, and we asked a question to determine if the event meets the Criterion-A definition of a traumatic event; we used the primary care PTSD screener questions (21) to assess PTSD symptoms related to the PMIE. We did this because of research that has shown that Criterion-A events can entail moral injuries (3) and to explore possible PTSD as a comorbid problem among individuals exposed to PMIEs. The research version of the MIOS used in Stage II and III allowed those without a PMIE to answer the PTSD screener questions and to fill out the MIOS with a most currently distressing stressor in mind to test the linkage between PMIE exposure and MIOS scores.

On the second page of the MIOS, we asked respondents to write out the worst and most currently distressing PMIE if they were comfortable doing so. This was followed by the preliminary 34 MIOS items, listed in random order. The instruction was: “Keeping this worst event in mind, please read each of these statements and circle one of the numbers to the right to indicate how much you would agree with the statement in the past month.” The response options were Likert-type, according to degree of agreement. We asked respondents to judge the global impact of the MIOS items on a Likert-type scale between 0 (*not at all*) to 6 (*extremely*) in terms of how much these experiences made it hard for respondents to take care of themselves (e.g., do pleasurable things, exercise, eat properly), and whether they were effective in their job, in school, seeking employment, or getting along with other people.

## Stage II: Examining Factor Structure and Item Trimming

The 34-item version of the MIOS was administered to Veterans and/or active-duty SMs in each participating country (Canada had two sites). All participants were different from the participants in Stage I. Analyses entailed an examination of the factor structure of the MIOS, using exploratory factor analyses (EFA). The Canada sample was used as the reference group because these were the first Stage II data collected. This was followed by confirmatory factor analysis (CFA) derived from the EFA model, also with the Canada sample, and cross-national multigroup invariance testing of the model. Finally, we report the interitem and item-total correlations, and internal consistency reliability of the trimmed scale.

### Methods

#### Participants

*Canada* (non-clinical sample). Canadian Armed Forces (CAF) Veterans were recruited to participate in a 30-min online survey comprising the MIOS and a series of additional psychological, social, and physical well-being measures as part of a larger study exploring Veteran well-being. They were recruited *via* word of mouth, email distribution through professional and Veteran group networks, participant recruitment websites, and social media. Research Electronic Data Capture (REDCap) was used to collect data. This research was approved by the Western University Health Sciences Research Ethics Board. Participants included 533 Veterans (71% men, mean age = 51.87 years [SD = 9.77]). Three hundred and sixty-six participants (68.7%) endorsed a PMIE. Of those who endorsed a PMIE, 49.7% endorsed a MI-Self, 71.0% endorsed observing a MI-Other based on observation of a transgression, and 82.5% were directly impacted from an MI-Other. The most common single type was direct impact from MI-Other (10.4%).

*United States* (non-clinical sample). Three hundred sixty-three Veterans participated in an online survey study conducted by Qualtrics comprising the 34-item MIOS. Of the 360 Veterans who participated in the study, 73.6% were men; ages ranged from 20 to 79 (*M* range = 50–59). Seventy-eight percent endorsed a PMIE [39% endorsed each type of PMIE (MI-self, MI-Other, and MI-O being directly impacted by another's transgression)]. Of those that only endorsed a single type, the most common was MI-self (11%). The research was approved by the IRB at the VA Boston Healthcare System.

*Canada* (Ottawa; clinical sample). Two hundred thirty-nine individuals with a treatment history of operational stress injuries participated in an online survey study using the 34-item MIOS. Of the 239 Veterans who participated in the study, 74.8% were men; the age range was 20–79 years (*M* range = 50–59). 89.9% of participants endorsed a PMIE: 51% endorsed an MI-Self, 74.6% endorsed observing an MI-Other, and 79.6% endorsed being directly impacted by a MI-Other. The most common single type was direct impact from MI-Other (9%). The research was approved by the Royal Ottawa Research Ethics Board.

*United Kingdom* (non-clinical sample). Two hundred sixty-four Veterans from the United Kingdom (UK) participated in an online survey study of the 34-item MIOS. Of the 264 Veterans who completed the MIOS, 67% were men; the mean age range was 51–60 years. All participants reported a PMIE (65.9% MI-Self; 64.4% observing a MI-Other, and 70.1% directly impacted by a MI-Other). MI-S was the most common single type (11.7%). The research was approved by the Combat Stress Research Committee.

*Australia* (non clinical sample). One hundred eighteen Defense members and Veterans participated in a survey study using the 34-item MIOS. Mean age range was 40–49 years. The MIOS was administered to participants either online or in person; 65.9% of participants identified as male. Of those who endorsed a PMIE, 55% endorsed a MI-Self, 79% endorsed observing a MI-Other, and 87% endorsed being directly impacted by an MI-Other. The most common single PMIE was the latter (12%). The research was approved by the Departments of Defense and Veterans' Affairs Human Research Ethics Committee.

#### Data Analytic Strategy

To investigate the dimensionality of the preliminary 34-item MIOS, we conducted an EFA with the Canadian sample using SPSS Version 26.0 (22)?. All participants completed at least 80% of the MIOS; we used pairwise deletion to handle missing data. Adequacy of data for factor analysis was measured using the Kaiser–Meyer–Olkin (KMO) measure of sampling adequacy, with values above 0.60 reflecting suitability for analysis (23), and Bartlett's test of sphericity (24), with statistically significant values indicating that item correlations are significantly different from zero. We used principal axis factoring (PAF) with direct oblimin rotation. Items were retained based on theory, consideration for item redundancy, and a cut-off value of 0.30 (25). Parallel analysis (26), very simple structure (27), Scree plots, and theory were considered to determine the number of factors to extract.

Using the Canada (Ottawa), US, UK, and Australian samples we conducted CFAs, with MPlus Version 8.0 (28). Sample sizes of at least 200 are recommended for CFA (29). The maximum likelihood estimator was used, and missing data were estimated using the full-information maximum likelihood. We used root mean square error of approximation (RMSEA), comparative fit index (CFI), and the Tucker-Lewis index (TLI) to evaluate model fit. Values of 0.06 reflected good fit, 0.07–0.08 acceptable fit, 0.08–0.10 marginal fit, and >0.10 poor fit; also, CFI and TLI values of >0.95 reflected excellent model fit and 0.90–0.95 indexed acceptable fit (30).

To evaluate cross-national invariance of MIOS scores, a series of multi-group confirmatory factor analytic (MGCFA) models were tested; the US and UK samples were each compared to the Canadian (Ottawa) sample. Three levels of invariance were tested: configural (i.e., number of factors is equivalent across groups), metric invariance (i.e., factor loadings are equivalent across groups), and scalar invariance (i.e., intercepts are equivalent across groups). If scalar invariance is satisfied, latent means can be reliably compared across groups; otherwise, intercept constraints can be freed sequentially to examine partial scalar invariance (31). Nested MGCFA models were compared using χ^2^, CFI, and RMSEA difference tests. CFI difference values less than or equal to 0.01, RMSEA difference values less than or equal to 0.01, and non-significant χ^2^ difference tests indicate that invariance is satisfied (31, 32).

Due to the small sample size (*n* = 118), we evaluated the invariance of the Australian sample compared to the Canadian sample using multiple indicators, multiple causes [MIMIC; (33, 34)] modeling, which does not require large sample sizes (35). Using MIMIC modeling, we tested for invariance across item intercepts and factor means. The covariate, country, was regressed onto the MIOS factors. If these coefficients are non-significant, then cross-national invariance of the factors is satisfied. In addition, we regressed country onto each item and fixed the direct effects to zero; following this, we examined the modification indices to determine whether any of the item intercepts were noninvariant (35). Where no modification indices emerged, we concluded that cross-national invariance of the intercepts was satisfied.

### Results

#### Exploratory Factor Analysis

We determined that the Canadian sample was suitable for an EFA because we found a KMO index of 0.96 and Bartlett's test of sphericity was significant, $\chi_{\left(561\right)}^{2}$ = 12,789.11, *p* < 0.001. When all initial 34 items were included, initial Eigenvalues and parallel analysis suggested that five factors should be retained (see Supplementary Figure 1). However, only one item loaded onto Factor 5, three items loaded onto Factor 4, and four items loaded onto Factor 3 without cross-loadings. In addition, the Scree plot and very simple structure indicated that two factors should be retained. A two-factor solution was consistent with theory and how we approached content development, namely that MIOS items would describe the outcomes unique to a MI-Self experience (we called this Factor 1, *Shame-Related; SR*), and uniquely applicable to MI-Other experiences (we called this Factor 2, *Trust-Violation-Related; TVR)*. An EFA using the 34 items found that Factors 1 and 2 accounted for 44.64% and 6.02% of the variance across items, respectively. The two factors correlated at 0.47. The factor loadings for the Canadian Stage II study for the preliminary 34-item scale are presented in Supplementary Material.

Next, we sought to reduce the scale to a parsimonious number of items while ensuring that content validity was maintained across the two factors. We sequentially removed items based on a combination of the following empirical and theoretical reasons: (1) factor loadings below 0.30; (2) cross-loadings exceeding 0.30; (3) maintaining coverage of all domains of impact; and (4) the redundancy of items. First, three items were removed due to low communalities. Next, five items were removed due to substantial content overlap (e.g., “I blame myself” was kept, “I feel guilty about what happened” was dropped). Next, three items were removed due to cross-loadings. Finally, nine items with some content overlap were removed from Factor 1 to maintain an approximately equal number of items across the factors (the items that comprise the SR and TVR subscales are presented in Table 2).

**Table 2:** Pattern matrix factor loadings for 14-item moral injury outcome scale.

**Item**	**Shame-related outcomes**	**Trust violation-related outcomes**
I am not the good person I thought I was	**0.91**	−0.13
I feel like I don't deserve a good life	**0.74**	0.02
I keep myself from having success	**0.73**	0.02
People would hate me if they really knew me	**0.71**	0.07
I have lost pride in myself	**0.67**	0.15
I blame myself	**0.60**	−0.01
I cannot be honest with other people	**0.55**	0.09
I have lost faith in humanity	−0.11	**0.87**
I lost trust in others	−0.05	**0.82**
I have trouble seeing goodness in others	−0.01	**0.77**
I am angry all the time	0.18	**0.62**
I am disgusted by what happened	0.02	**0.49**
People don't deserve second chances	0.09	**0.38**
I no longer believe there is a higher power	0.05	**0.30**

*Bolded values represent loadings ≥0.30*.

We then conducted an EFA using the final 14-item MIOS. The KMO index (0.93) and Bartlett's test of sphericity [$\chi_{\left(91\right)}^{2}$ = 3,302.71, *p* < 0.001] indicated that the data were suitable for EFA. Factor 1 accounted for 42.15% of variance among items, while Factor 2 accounted for 5.75% of variance. The two factors were correlated at 0.74, indicating that they represent unique but associated elements of MI (with 55% shared variance). Item loadings were strong (see Table 2), ranging from 0.30 (“I no longer believe there is a higher power”) to 0.91 (“I am not the good person I thought I was”). Although the loading for “I no longer believe there is a higher power” was weaker than the remaining loadings, it was important to include this item to preserve the content domain reflecting beliefs about life meaning and purpose.

#### Descriptive Results

Table 3 shows the means, standard deviations, and Cronbach's alphas for the 14-item MIOS for each sample. Internal consistency values were acceptable, ranging from 0.85 (Ottawa) to 0.90 (US and Canada) for the 14-item MIOS. The Stage II bivariate correlations between SR and TVR subscale scores were 0.66, 0.51, 0.68, 0.67, and 0.50 in the Canada, Ottawa, US, UK, and Australian study groups, respectively.

**Table 3:** Descriptive statistics for 14-item moral injury outcome scale.

**Variable**	***M***	***SD***	**α**
**Canada**			
MIOS total score	25.31	11.38	0.90
Shame-related outcomes	11.28	6.64	0.88
Trust violation outcomes	14.03	5.85	0.81
**Canada (Ottawa)**			
MIOS total score	27.32	9.08	0.85
Shame-related outcomes	11.98	5.75	0.85
Trust violation outcomes	15.34	4.69	0.72
**United States**			
MIOS total score	25.14	11.36	0.90
Shame-related outcomes	11.36	6.82	0.90
Trust violation outcomes	13.78	5.58	0.78
**United Kingdom**			
MIOS total score	32.87	10.54	0.89
Shame-related outcomes	16.29	6.20	0.86
Trust violation outcomes	16.58	5.35	0.79
**Australia**			
MIOS total score	27.74	10.42	0.88
Shame-related outcomes	12.17	6.35	0.86
Trust violation outcomes	15.56	5.52	0.81

Item-level descriptive statistics, inter-item, and item-total correlations for the 14-item MIOS for the Canadian sample are shown in Table 4 (other Stage II consortia results are presented in Supplementary Material). Although there is no consensus regarding optimal inter-item correlation range, Clark and Watson (15) suggested that the average item-total correlation should range between 0.15 to 0.50. The average inter-item correlation for the MIOS was 0.40, which provides evidence that the items represent the same underlying construct, but they are not redundant. Additionally, all item-total correlations reached a recommended cutoff of 0.30 (15, 36), but were not so high as to suggest that the measure lacks breadth of content [(37); uncorrected *r* range = 0.42–78, corrected *r* range = 0.30–73].

**Table 4:** Moral injury outcome scale descriptive statistics and inter-item/item-total correlations—Canada.

**Item**	***M (SD)***	**1**	**2**	**3**	**4**	**5**	**6**	**7**	**8**	**9**	**10**	**11**	**12**	**13**	**14**
1. I blame myself	1.94 (1.29)	1													
2. People would hate me if they really knew me	1.54 (1.27)	0.45	1												
3. I feel like I don't deserve a good life	1.33 (1.21)	0.47	0.60	1											
4. I keep myself from having success	1.71 (1.18)	0.47	0.52	0.58	1										
5. I am not the good person I thought I was	1.51 (1.16)	0.49	0.65	0.60	0.57	1									
6. I have lost pride in myself	1.89 (1.32)	0.45	0.55	0.58	0.64	0.65	1								
7. I cannot be honest with other people	1.41 (1.25)	0.33	0.53	0.42	0.43	0.50	0.49	1							
8. I am angry all the time	1.93 (1.25)	0.43	0.48	0.48	0.47	0.50	0.55	0.37	1						
9. I have lost faith in humanity	2.22 (1.26)	0.32	0.46	0.44	0.37	0.38	0.50	0.31	0.60	1					
10. I have trouble seeing goodness in others	1.91 (1.20)	0.27	0.49	0.44	0.43	0.40	0.49	0.41	0.57	0.61	1				
11. People don't deserve second chances	1.31 (1.02)	0.15	0.28	0.31	0.27	0.30	0.30	0.28	0.29	0.30	0.38	1			
12. I am disgusted by what happened	2.45 (1.21)	0.28	0.31	0.30	0.26	0.26	0.31	0.25	0.41	0.35	0.33	0.31	1		
13. I no longer believe there is a higher power	1.87 (1.32)	0.17	0.25	0.19	0.19	0.22	0.21	0.20	0.23	0.30	0.28	0.18	0.18	1	
14. I lost trust in others	2.40 (1.24)	0.36	0.41	0.40	0.47	0.40	0.50	0.39	0.58	0.63	0.59	0.33	0.43	0.23	1
Total MIOS	1.82 (0.82)	0.54	0.70	0.68	0.66	0.69	0.73	0.57	0.70	0.65	0.66	0.41	0.46	0.30	0.66
		(0.61)	(0.75)	(0.73)	(0.72)	(0.74)	(0.78)	(0.64)	(0.75)	(0.71)	(0.72)	(0.49)	(0.53)	(0.42)	(0.72)

*All correlations significant at p < 0.001. Uncorrected item-total correlations in brackets (else are corrected item-total correlations)*.

#### Confirmatory Factor Analyses

Using the Ottawa sample, the 14-item two-factor model fit the data well, $\chi_{\left(76\right)}^{2}$ = 154.56, *p* < 0.001, CFI = 0.923, TLI = 0.907, RMSEA = 0.066 (90% CI = 0.051, 0.081). Factor loadings were strong, ranging from 0.30 (“I am disgusted by what happened”) to 0.79 (“I feel like I don't deserve a good life”), and the factors were significantly correlated at 0.65. In the US sample, the two-factor model also fit the data well: $\chi_{\left(76\right)}^{2}$ = 179.01, *p* < 0.001, CFI = 0.954, TLI = 0.944, RMSEA = 0.061 (90% CI = 0.050, 0.073; see Supplementary Figure). All items loaded significantly onto their respective factors, ranging from 0.32 (“I no longer believe there is a higher power”) to 0.81 (“I feel like I don't deserve a good life”), and the factors were significantly correlated at 0.77. Finally, the UK model fit the data well: $\chi_{\left(76\right)}^{2}$ = 171.14, CFI = 0.928, TLI = 0.913, RMSEA = 0.069 (90% CI = 0.055, 0.083). Loadings were strong for the UK sample, ranging from 0.46 (“People don't deserve second chances”) to 0.77 (“I am not the good person I thought I was”), and the factors were significantly correlated at 0.78. Although the correlations were high, they do not exceed the cutoff values of 0.80 to 0.85 and are therefore not considered redundant (35).

#### Cross-National Invariance

The US—Ottawa configural model fit the data well, $\chi_{\left(152\right)}^{2}$ = 331.72, *p* < 0.001, CFI = 0.944, TLI = 0.933, RMSEA = 0.063 (90% CI = 0.053–0.072) indicating that the number of factors was consistent across countries (see Table 5). Factor loadings were also equivalent between the US and Ottawa samples [Chi-square, CFI, and RMSEA difference tests demonstrated no significant differences in fit between the metric and configural models, Δ$\chi_{\left(12\right)}^{2}$ = 12.39, *p* > 0.05, ΔCFI = 0.000, ΔRMSEA = 0.003]. Next, the RMSEA difference test revealed that the scalar invariance model was not significantly different from the metric model, ΔRMSEA = 0.004. However, both the chi-square and CFI difference tests surpassed the cut-off values, Δ$\chi_{\left(12\right)}^{2}$ = 49.20, *p* < 0.01, ΔCFI = 0.012. When we freed the intercept for the item “I feel like I don't deserve a good life,” we achieved partial scalar invariance according to CFI and RMSEA difference tests, Δ$\chi_{\left(11\right)}^{2}$ = 34.14, *p* < 0.01, ΔCFI = 0.007, ΔRMSEA = 0.002. We compared latent means and found no significant differences between the US and Ottawa in SR Outcomes (Δ*m* = 0.13, *p* = 0.097), but Canada scored higher than the US on latent TVR Outcomes (Δ*m* = 0.20, *p* = 0.002).

**Table 5:** Cross-national invariance fit indices.

**Model**	**χ^2^ (*df*)**	**CFI**	**TLI**	**RMSEA**	**RMSEA 90% CI**
**US and Canada (Ottawa)**					
Configural model	331.72 (152)***	0.944	0.933	0.063*	0.053, 0.072
Metric model	344.11 (164)***	0.944	0.938	0.060*	0.051, 0.069
Scalar model	393.30 (176)***	0.932	0.930	0.064**	0.056, 0.073
Partial scalar model	378.25 (175)***	0.937	0.934	0.062*	0.054, 0.071
**UK and Canada (Ottawa)**					
Configural model	326.14 (152)***	0.925	0.910	0.068**	0.057, 0.078
Metric model	340.51 (164)***	0.924	0.916	0.065**	0.056, 0.075
Scalar model	406.54 (176)***	0.901	0.897	0.072***	0.063, 0.081
Partial scalar model	354.55 (175)***	0.915	0.911	0.066**	0.056, 0.075

***p < 0.001.

**p < 0.01.

*p < 0.05.

*CFI, comparative fit index; TLI, Tucker-Lewis Index; RMSEA, Root mean square error of approximation; CI, confidence interval*.

The UK—Ottawa configural model also showed strong fit to the data, $\chi_{\left(152\right)}^{2}$ = 326.14, *p* < 0.001, CFI = 0.925, TLI = 0.910, RMSEA = 0.068 (90% CI = 0.057–0.078). According to the chi-square, CFI, and RMSEA difference tests, the metric model did not differ significantly from the configural model, Δ$\chi_{\left(12\right)}^{2}$ = 14.37, *p* > 0.05, ΔCFI = 0.001, ΔRMSEA = 0.003, indicating that factor loadings were equivalent across countries. Although the RMSEA difference test indicated that scalar invariance was satisfied, ΔRMSEA = 0.007, the chi-square and CFI difference tests revealed that scalar invariance was not met, Δ$\chi_{\left(12\right)}^{2}$ = 66.03, *p* < 0.01, ΔCFI = 0.023. After freeing the intercept for “People don't deserve second chances”, partial scalar invariance was satisfied, Δ$\chi_{\left(11\right)}^{2}$ = 14.04, *p* > 0.05, ΔCFI = 0.009, ΔRMSEA = 0.001. Therefore, mean differences were calculated, and UK scored significantly higher than Canada on latent SR Outcomes (Δ*m* = 0.59, *p* < 0.001), as well as latent TVR outcomes (Δ*m* = 0.16, *p* = 0.020).

#### MIMIC Model

First, we regressed country (Australia and Canada) onto both MIOS factors. This model fit the data well, $\chi_{\left(88\right)}^{2}$ = 184.15, *p* < 0.001, CFI = 0.940, TLI = 0.928, RMSEA = 0.055 (90% CI = 0.044–0.067). As expected, the covariate country did not have a significant effect on SR (β = 0.03, SE = 0.086, *p* = 0.688) or TVR outcomes (β = 0.008, SE = 0.079, *p* = 0.919). Next, we regressed country onto each item and fixed the direct effects to zero. Modification indices were <3.12, indicating cross-country fit (35).

### Finalization of the MIOS

Based on feedback from clinicians and an evaluation of consortium members about the MIOS scale, we finalized the formatting. The final research version of the MIOS has two pages. The first page entails an assessment of exposure to three types of PMIEs, defined as events that went against the person's moral code or values [doing something or failing to do something, observing someone else acting or failing to act, or being directly impacted by someone else (or people) acting or failing to act]. We retained the primary care PTSD screener items (21). The second page of the research version of the MIOS assesses the final set of 14 items determined from Stage II analyses, all indexed to the PMIE that is the worst and most currently distressing. The time frame for ratings is the last month. Scale scores are indexed by the sum of item scores. The final research version and the brief clinical versions of the MIOS are presented in the Supplementary Material (the MIOS is a public domain scale), along with scoring instructions. A brief version of the MIOS for clinical care and epidemiological studies is also presented in the Supplementary Material.

At the end of the MIOS, we decided to use the Brief Inventory of Psychosocial Functioning [B-IPF; (38)] to assess the functional impact of the MIOS symptoms endorsed across seven domains (romantic relationships, relationships with children, family relationships, friendships, work, training/education, and day to day activities). The B-IPF has high internal consistency and adequate test-retest reliability (38). The instructions embedded in the MIOS are: “Please write in a number for each item below that represents how much these experiences have made it hard for you to function in each of the following areas (if not applicable, use N/A)” The MIOS is designed to assess symptom burden (severity), but it is also designed to identify cases that have clinically significant functionally impairing symptoms. This will require future diagnostic utility studies, using signal detection analyses, with severe functional impairment as the criterion.

## Stage III: Test-Retest Reliability and Convergent Validity

### Predictions

We predicted that MIOS total *and* subscale scores would be strongly associated with measures of constructs that have been hypothesized to be overlapping parts of the MI syndrome or that are similar to the domains of impact generated in Stage I. These are: (1) *depression*. Litz et al. (1) predicted that MI would be associated with dysphoria, hopelessness, and self-esteem deficits; (2) *PTSD*. Litz et al. (1) predicted that individuals suffering because of exposure to PMIEs would experience intrusive recall and avoidance, as well as disinterest and detachment; and (3) *functional impairments*. Several domains of impact entail functional deficits and we have posited that the dividing line between moral distress and injury is chiefly the degree of functional impact related to the PMIE.

We had two sets of predictions of variables that would distinguish the MIOS SR and TVR subscales, namely: (1) that relative to MIOS TVR subscale scores, SR subscale scores would be more strongly correlated with reports of the *moral emotions of guilt/shame and religious and spiritual beliefs and practices*. The latter hypothesis is that personal transgressive acts are more likely to be morally injurious because they entail questions about right and wrong and good and evil (39); and (2) that relative to MIOS SR subscale scores, TVR scores would be more highly correlated with reports of the moral emotion of *anger and anger-related problems*. The assumption is that TVR MI entails externalizing, relative to SR MI. We also examined the association between the MIOS and the EMIS-M (5).

Finally, to investigate the validity of the assumption that MI is a PMIE-linked problem and the validity of the event-linkage aspect of the MIOS (i.e., indexing symptoms to a putative worst and most currently distressing PMIE), we ensured that 70 US participants (see below) who did not endorse a PMIE would be allowed to participate in the survey (MIOS ratings were instead indexed to a worst and most currently distressing life stressor). We predicted that individuals who did not endorse a PMIE would have substantially lower MIOS total and subscale scores, relative to participants who endorsed a PMIE.

### Methods

#### Procedure

We report studies conducted in the US, Australia, and Israel (all participants were different from the participants in Stage I and II and all samples were non-clinical). For the US study, the final 14-item MIOS was administered along with the measures described below (and a demographic and military service characteristics form) in an online 30-min survey study of US Veterans conducted by Qualtrics. Qualtrics recruited participants *via* various web-based sources, including website intercept recruitment, member referrals, targeted email lists, gaming sites, customer loyalty web portals, permission-based networks, and social media. Qualtrics then administered the survey to a nationally representative sample of 420 US military Veterans (*n* = 317) and active-duty SMs in the US military (*n* = 103), who had been deployed to a post-9/11 conflict. Participants were also required to have experienced a PMIE to complete the survey. However, Qualtrics was asked to accrue a subset of US participants (*n* = 70) who had not experienced a PMIE to conduct planned comparative analyses of MIOS scores between those who had experienced a PMIE vs. those who had not. For the Australia study, the measures were administered in an online survey of current and ex-serving members of the military aged 18 years or older who endorsed a PMIE during military service. Participants were recruited through advertising in social media and through Defense, the national veterans counseling service and ex-service organizations. There were 91 participants (34 current serving and 57 ex-serving members). For the Israel study (*n* = 111), the MIOS was translated to Hebrew by a coauthor and then back-translated into English by another author, both native English and Hebrew speakers; each agreed that the original version and the back translation were similar, and no additional modifications were required. Measures were administered in an online survey of current and ex-serving Israeli members of the military. Recruitment entailed advertisements in combat Veteran websites and academic centers. For the Israel study, inclusion criteria were at least 20 years of age, currently or formerly serving in a combat unit of the Israeli Defense Forces, and service in the last 20 years.

The order of survey scales was randomized in two unique iterations that participants were assigned to at random, but both iterations included the MIOS as the first scale that participants were required to complete. All state-based measures were indexed to the past month. For the US study only, participants were required to answer all questions in a measure before moving on in the survey *via* the Qualtrics “Forced Response” option. This method prevented participants from continuing without answering a missed question, which has been shown not to affect the reliability of online surveys (40). Therefore, there were no missing responses in the final US dataset, except for one question that asked participants to write-out their PMIE if they felt comfortable. Only survey completers were included in the final dataset for each country.

After the other Stage III data were collected, our Israeli partners examined the test-retest reliability of the final MIOS. The Ruppin Academic Center IRB approved the study. The same inclusion criteria as the Israeli Stage III study were applied and the demographics of the study group were similar. Eighteen SMs and Veterans completed the MIOS twice, a week apart.

#### Measures

##### Tests of Convergent Validity

###### Mental and Behavioral Health.

Depression symptom severity was measured with the nine-item Patient Health Questionnaire [PHQ-9; (41)]. Participants endorsed items on a 4-point frequency scale (0 = *not at all* to 3 = *nearly every day*) about their depressive symptoms in the last 14 days. Item responses were summed, with higher scores reflecting greater severity of depression symptoms. The PHQ-9 is the most used depression measure and has very strong internal consistency reliability and validity [α = 0.89; (42)]. PTSD symptom severity over the past 30 days was assessed with the 20-item PTSD Checklist for the DSM-5 [PCL-5; (43)]. Items were endorsed on a 5-point scale (0 = *not at all* to 4 = *extremely*). Item responses were summed, with higher scores representing greater PTSD symptom severity. The PCL-5 has been shown to be highly reliable and valid [α = 0.94; (43)]. Functional impairment was assessed with the B-IPF (38) at the end of the MIOS.

###### Moral Emotions.

State guilt and shame were assessed with the 10-item version of the State Guilt and Shame Scale [SGSS; (44)]. Participants endorsed items on a 5-point scale (1 = *not feeling this way at all* to 5 = *feeling this way very strongly*). Item responses were summed to create a total score for state guilt and shame. The SGSS has been shown to be reliable and valid [α = 0.85; (44)]. We also used a short 16-item version of the Trauma-related Guilt Inventory [TRGI; (45)]. The TRGI was developed to assess guilt feelings and attitudes about a specific traumatic event. The brief TRGI yields three averaged subscale scores: Hindsight-bias/responsibility, assessing self-blame and beliefs the event should have been prevented (seven items; Cronbach's α = 0.89); Wrongdoing, assessing perceived transgression in behavior, thoughts, and emotions (five items; Cronbach's α =0.73); and Lack of Justification, assessing the inability to justify actions (four items; Cronbach's α = 0.83). The TGRI scale has high internal consistency and test-retest reliability (45). Finally, we administered the 5-item Dimensions of Anger Reactions [DAR-5; (46)] as a brief measure of state anger. Participants endorsed items on a 5-point scale (1 = almost none of the time to 5 = all or almost all of the time). Item responses were summed to create a total anger score, with higher scores representing greater anger levels. The DAR-5 has been shown to have convergent validity and is highly reliable [α = 0.97; (46)].

###### Religion and Spirituality.

Religious and spiritual struggles were assessed with an eight-item version of the Religious and Spiritual Struggles Scale [RSS; (47)]. Participants endorsed items on a 5-point scale (1 = *not at all* to 5 = *a great deal*). Item responses were summed to create a total score for religious and spiritual struggles, with higher scores indicating greater struggles. The RSS has been found to be reliable and has good convergent, discriminant, and predictive validity [α = 0.87; (47)].

###### Moral Injury.

To assess MI as an outcome, we used the 17-item Expressions of Moral Injury Scale—Military Version [EMIS-M (5)]. Participants endorsed items on a 5-point scale (1 = *strongly disagree* to 5 = *strongly agree*). Item responses were summed to create a total score.

### Results

#### Descriptive Statistics

The sociodemographic and military service characteristics of the US, Australian, and Israeli groups are shown in a Table in the Supplementary Material. In the US study, the group was predominantly white men, with a modal age range of 30–39 (to enhance anonymity, we used age rages rather than age), and ~24% were active-duty SMs. All US participants served in the Iraq or Afghanistan Wars (primarily deployed between 2001 and 2010) and the majority had combat arms duty while serving, which means that the majority participated in tactical ground combat and likely entailed multiple exposures to high magnitude warzone stressors and potentially traumatizing and morally injurious events. This is atypical for US Veteran survey studies that generally have majorities of service support personnel with substantially less combat exposure (4). By contrast, the Australia study group was substantially older [modal age range = 40–59 [17.3% were 60–79)]; 25% were never deployed to a warzone and, although 54.3% endorsed deploying to a “warlike” context, which unfortunately leaves unspecified the types of roles within that context, 79% reported being deployed in their careers in peacekeeping, humanitarian, and border protection missions, which are missions typically associated with bearing witness to others' transgressions and grotesque harm to others (48). Ninety-one percent of the Israeli participants were Veterans, the majority were male (75%), and 90% were in the 20–29 age range (substantially younger than both other cohorts).

Means and standard deviations for all scales for the PMIE-endorsers in all studies are reported in Table 6. Internal consistency values of the MIOS were strong across all samples, with Cronbach's alphas ranging from 0.88 (TVR) to 0.95 (total) in the US sample, 0.83 (TVR) to 0.89 (total) in the Australian sample, and 0.83 (TVR) and 0.90 (total) in the Israeli sample. Because the sample size was sufficient, we conducted a CFA to confirm the two-factor structure of the MIOS in the US Phase III sample (see Supplementary Material).

**Table 6:** MIOS total and subscale descriptives and correlations across all phase III sample PMIE-endorsers.

**Variable**	**US**					**Israel**					**Australia**				
	***N***	***M* (SD)**	**1**	**2**	**3**	***N***	***M* (SD)**	**1**	**2**	**3**	***N***	***M* (SD)**	**1**	**2**	**3**
1. MIOS Total Score	350	33.59 (13.37)	–			71	14.55 (9.28)	–			87	27.3 (9.98)	–		
2. MIOS Shame Subscale	350	16.51 (7.28)	0.966**	–		71	5.96 (5.2)	0.886**	–		87	11.8 (6.07)	0.864**	–	
3. MIOS Trust Subscale	350	17.08 (6.62)	0.958**	0.851**	–	71	8.59 (5.25)	0.889**	0.575**	–	87	15.49 (5.64)	0.840**	0.451**	–
PCL-5	349	50.05 (19.41)	0.729**	0.705**	0.698**	71	20.01 (16.48)	0.574**	0.587**	0.433**	84	58.95 (19.22)	0.631**	0.496**	0.567**
PHQ-9	350	15.35 (6.59)	0.619**	0.619**	0.569**	71	2.51 (3.84)	0.486**	0.475**	0.388**	79	11.76 (6.74)	0.520**	0.406**	0.458**
B-IPF	350	30.50 (9.06)	0.717**	0.690**	0.689**	69	22.96 (24.84)	0.441**	0.327**	0.455**	87	53.40 (26.69)	0.636**	0.569**	0.511**
TRGI	350	2.09 (0.68)	0.301**	0.363**	0.209**	71	1.36 (0.78)	0.403**	0.536**	0.181	77	1.39 (0.77)	0.379**	0.580**	0.026
SSGS	350	34.77 (10.24)	0.696**	0.732**	0.602**	71	19.44 (8.75)	0.687**	0.673**	0.546**	84	22.54 (9.22)	0.541**	0.660**	0.228*
RSS Scale	350	3.42 (0.98)	0.685**	0.707**	0.607**	71	2.20 (1.01)	0.667**	0.574**	0.610**	72	18.54 (8.26)	0.513**	0.457**	0.386**
DAR-5	350	16.28 (5.41)	0.711**	0.684**	0.685**	71	11.20 (4.62)	0.664**	0.573**	0.606**	82	12.40 (6.00)	0.634**	0.538**	0.525**
EMIS-M Self Subscale	350	30.91 (8.43)	0.744**	0.745**	0.684**	71	15.30 (5.97)	0.782**	0.755**	0.633**	70	23.17 (7.43)	0.686**	0.588**	0.547**
EMIS-M Other Subscale	350	29.13 (7.15)	0.643**	0.587**	0.654**	71	19.75 (8.63)	0.718**	0.587**	0.687**	70	28.19 (7.44)	0.479**	0.249*	0.558**

*B-IPF, Brief Inventory of Psychosocial Functioning; DAR-5, Dimensions of Anger Reactions-5; EMIS-M, Expressions of Moral Injury Scale—Military Version; MIOS, Moral Injury Outcome Scale; PCL-5, PTSD Checklist for DSM-5; PMIE, Potentially Morally Injurious Event; PHQ-9, Patient Health Questionnaire-9; RSS, Religious and Spiritual Struggles; SSGS, State Shame and Guilt Scale; TRGI, Trauma-Related Guilt Inventory*.

*p < 0.05.

**p < 0.01.

The types of PMIEs endorsed and the PTSD screener results for the PMIE-endorsers for each study (and non-endorsers for the US and Israeli studies) are presented in a Supplementary Table. In the US study, 73.1% of PMIE endorsers reported at least one PMIE related to the self, 80% endorsed at least one PMIE related to another, and 84.3% endorsed at least one betrayal event. When asked to endorse the worst and most currently distressing PMIE (using a forced choice), 45.7% endorsed a self-transgression (32.9 and 21.4% endorsed PMIE-other and PMIE-betrayal, respectively). In addition, 82.2% of the US participants' worst and most currently distressing PMIEs met Criterion-A (the PMIE was reported to involve actual or threatened death, serious injury, or sexual violence), and 52.1% of PMIE-endorsers that met Criterion-A endorsed 4 or 5 PTSD screener items (26.4% and 25.7%, respectively), and thus likely had clinically significant PTSD symptoms as a putative result of the PMIE or the context in which the PMIE occurred; 4/5 screener items endorsed is the most diagnostically efficient; 5/5 is the most specific (21) [in this group, the Mean PCL-5 score was 55.72 (SD = 17.7)]. Yet, in the US sample, there were no differences in the percentage of PMIE-endorsers whose event was not a Criterion-A trauma (which formally eliminates the possibility of PTSD caseness) who endorsed 4 or 5 PTSD screener items, relative to those who endorsed Criterion-A [the Mean PCL-5 score for this subgroup was 53.90 (SD = 19.45); mean difference (95% CI): 1.82 (−7.66, 11.30), *p* < 0.695]. In the Australia study, 45.1% of participants endorsed a history of exposure to at least one PMIE-self event, 74.7% endorsed at least one MI-other event, and 79.1% endorsed at least one PMIE-betrayal event. When asked to endorse the worst and most currently distressing PMIE, 83.9% endorsed PMIE-other or PMIE-betrayal (non-self-PMIEs). In addition, 60% of the Australia participants' worst and most currently distressing PMIEs met Criterion-A, and 56.3% endorsed 4 or 5 PTSD screener items. In the Israeli study, 63.4% of participants endorsed a history of exposure to at least one PMIE-self event, 60.6% endorsed at least one MI-other event, and 21.1% endorsed at least one PMIE-betrayal event. When asked to endorse the worst and most currently distressing PMIE, 38% endorsed PMIE-self, 39.4% a PMIE-other event, and 22.5 endorsed a PMIE-betrayal event. In addition, 38% of the Israeli participants' worst and most currently distressing PMIEs met Criterion-A, and 11% endorsed 4 screener items (none endorsed 5).

### Test-Retest Reliability

Bland-Altman Limits of Agreement [LOA; (49)] were calculated to assess test-retest reliability (*n* = 17). LOA use descriptive statistics for paired data to represent upper and lower boundaries of the middle 95% range of observed within-pair differences, centered around the mean within-pair difference. Confidence intervals (95%) are calculated around the upper and lower limits to improve inference beyond the sample. LOA are preferable to correlation analyses when determining test-retest reliability, as correlation analyses may conceal systematic bias (50). LOA uses an a priori determination of acceptable within-pair difference; for the MIOS we determined this to be ±14, which represents a within-pair difference of ±1 on all MIOS items. After removing an outlier, the LOA's and the upper and lower 95% CIs were −8.62 (−12.83 to −4.41) to 9.12 (4.91 to 13.34), which were within acceptable limits to establish test-retest reliability and the bias estimate was small β = 0.25.

#### Convergent Validity: MIOS Total and Subscale Scores

Table 6 depicts the Pearson correlations for PMIE endorsers between MIOS total and subscale scores and Stage III measures in the US, Australian, and Israeli samples. The intercorrelations of all variables for PMIE-endorsers and non-endorsers (only for US and Israel studies which had sufficient Ns) are presented in Supplementary Material. We used procedures developed by Meng et al. (51) to examine contrasts between correlated associations to test hypotheses. In each sample, as predicted, MIOS total and subscale scores were strongly correlated with measures of mental and behavioral health (PTSD, depression, and functional impairments). Although, in the US study, these correlations were substantially higher, *they were no higher than the correlations between the PCL-5 and the PHQ-9 and B-IPF* (Z-scores for these three contrasts were NS).

#### Convergent Validity: MIOS Subscale Scores

As can be seen in Table 7, as predicted, in each sample, MIOS SR subscale scores were more strongly correlated with the TRGI and the SSGS, relative to the TVR subscale. In the US sample, MIOS SR subscales scores were more correlated with RSS scores, relative to TVR subscale scores. Contrary to our prediction, across all samples, the SR and TVR subscales were equally correlated with the DAR scores.

**Table 7:** Convergent validity contrasts by MIOS shame-related and trust violation-related subscale scores.

**Variable**		**US**			**Israel**			**Australia**		
**Measure**	**Contrast type**	*Z*-score*	**95% confidence interval for difference**	***p*-Value**	***Z*-score**	**95% confidence interval for difference**	***p*-Value**	***Z*-score**	**95% confidence interval for difference**	***p*-Value**
TRGI	MIOS SR vs. TVR	5.69	0.11 0.23	<0.001	3.47	0.18 0.65	<0.001	5.76	0.42 0.85	<0.001
SSGS	MIOS SR vs. TVR	6.15	0.16 0.31	<0.001	1.6	−0.5 0.47	0.055	4.98	0.42 0.85	<0.001
RSS	MIOS SR vs. TVR	4.97	0.11 0.26	<0.001	0.42	−0.20 0.31	0.34	0.72	−0.16 0.36	0.236
DAR	MIOS TVR vs. SR	All *Z*-scores were non-significant								

*Z-scores and CIs are rounded to two decimals*.

*TRGI, Trauma-Related Guilt Inventory; SSGS, State Shame and Guilt Scale; RSS, Religious and Spiritual Struggles; DAR-5, Dimensions of Anger Reactions-5*.

*Formula for Z-score is provided by Meng et al. (51).

#### MIOS Score Differences Between PMIE Endorsers and Non-endorsers

As predicted, in the US study, the group that did not endorse a PMIE had significantly lower MIOS total and SR and TVR subscale scores (*M* = 20.90, SD=13.37; *M* = 9.92, SD = 6.86; *M* = 10.97, SD = 6.79, respectively) than the group who endorsed a PMIE [*M* = 33.58, SD = 13.37; *t*_(418)_ = −7.25, *p* < 0.001; *M* = 16.50, SD = 7.27; *t*_(418)_ = −6.97, *p* < 0.001; *M* = 17.07, SD = 6.62, *t*_(418)_ = −7.01, *p* < 0.001, respectively]. The magnitude of the differences in scores between these two groups was substantial, as indicated by very large Cohen's d effect sizes (0.91–0.95; the Australian study did not have enough non-PMIE endorsers to conduct this analysis). In the Israeli study, the group that did not endorse a PMIE had significantly lower MIOS total and SR and TVR subscale scores (*M* = 5.95, SD=6.80; *M* = 2.45, SD = 3.78; *M* = 3.50, SD = 3.78, respectively) than the group who endorsed a PMIE [*M* = 14.55, SD = 9.28; *t*_(109)_ = 5.13, *p* < 0.001; *M* = 5.96, SD = 5.20; *t*_(109)_ = 3.74, *p* < 0.001; *M* = 8.59, SD = 5.25, *t*_(109)_ = 5.39, *p* < 0.001, respectively]. These differences were also substantial (effect sizes 0.77 to 1.19). Moreover, there were substantial differences in B-IPF scores that were indexed to MIOS symptoms among PMIE-endorsers and non-endorsers in the US study [endorsers: 70.51, SD=25.36 vs. non-endorsers: 48.89 SD = 33.06; mean difference (95% CI): 21.63 (14.54, 28.71), *p* < 0.001] and the Israeli study [endorsers: 22.96, SD=24.84 vs. non-endorsers: 8.06 SD = 23.25; mean difference (95% CI): 14.91 (5.34, 24.28), *p* < 0.003]. This suggests that PMIE-endorsement is associated with markedly greater impairments indexed to MIOS items, relative to impairments indexed to MIOS symptoms indexed to a non-PMIE stressor.

## Discussion

There has been an explosion of interest in MI in healthcare, mental health, the media, in and outside the military and organizations that address the behavioral health needs of Veterans, and various scholarly and applied disciplines. Unfortunately, acceptance of the idea of MI has outpaced scientific knowledge, yet, in many contexts, the concept of MI is reified. This is particularly problematic because there are widely varying uses of the MI term, which is not surprising given that there has been no agreement about the boundary conditions of the MI syndrome. Existing empirical studies have also used imprecise terminology and have been hampered by a lack of a gold standard of measurement. In addition, treatments have been developed to putatively target MI, which is cart before the horse without a definition of the MI syndrome, a case definition, and a way to assess efficacy.

We aimed to redress these problems by using theory and multinational bottom-up phenomenological evaluations of the impact of exposure to PMIEs to operationalize the syndrome of MI into constituent domains of impact of PMIEs. We then used the definitions of the domains of impact (and components with each domain) to create a psychometrically sound measure of MI (indexed by reports of exposure to a worst and most currently distressing PMIE), that could be used in clinical and research settings to identify functionally impairing MI, and to track change. We generated content for the MIOS from multinational interviews with SMs and Veterans who were asked to describe how exposure to their worst PMIE changed their beliefs, emotions, and behaviors, as well as with mental and spiritual health care-providers asked to describe the problems and struggles of individuals with MI, ensuring strong cross-country content validity (albeit in English-speaking countries). The final 14-item MIOS was found to be highly reliable and had a robust two-factor structure, entailing SR and TVR items (7-items each). The MIOS also had partial scalar invariance across nations.

In Stage II and III, the correlations between the MIOS subscales were moderate, suggesting that SR and TVR are separable but related subconstructs of MI. However, the correlation was high in the US Stage III sample, suggesting that the subscales may have substantially less discrimination. A possible explanation is that for the US sample, current MI-related problems are a gestalt blend of SR and TVR symptoms among SMs and Veterans with direct combat roles and high combat exposure, very high rates of at least one self- *and* other-related PMIE, and high rates of PMIEs that were associated with life-threat or the loss of life (unlike individuals evaluated in Stage I and II, and unlike the other Stage III study groups). This hypothesis would be equally germane to other contexts [e.g., refugees who suffer chronic political violence and traumatic trust violations in their home country and who do things or fail to do things that violate their deeply held moral beliefs to survive passage to a putatively safer country; (52)]. It should be noted that in population studies of US Veterans, PTSD subclusters are also very highly correlated [e.g., in one study, the reexperiencing subcluster was correlated 0.795 with the negative alterations in cognitions and mood subcluster; (53)]. If additional research also shows that MIOS subscales are highly correlated among individuals with multiple exposures to traumas and both self- and other-PMIEs, MIOS total scores may be the only valid index of MI in these contexts (clinically, the recommendation would be to interview the person further to determine whether there is a pressing and most currently distressing event and domains of impact applicable to that event).

The convergent validity findings for MIOS total and subscale scores were consistently strong. Across Stage III studies, there were consistently large associations between indicators of mental and behavioral health and functional impairments and MIOS total and subscale scores. This is consistent with the theory that posits that some PTSD (e.g., reexperiencing, avoidance, detachment) and depression symptoms (dysphoria, hopelessness, anhedonia) are associated with exposure to any type of PMIE and are de facto aspects of the MI syndrome (1). As stated above, we purposely generated content for the MIOS that was distinct from the overlapping features of PTSD and depression that were endorsed by Stage I participants, and we assume that the resulting domains of impact, reflected in MIOS content, are core drivers of MI and will prove to be beneficial targets of treatment for functionally impairing MI.

The differential convergent validity predictions for the MIOS subscales were partially confirmed. Relative to the TVR subscale, SR subscale scores were consistently more strongly correlated with constructs that measure guilt and shame. And, in the US Stage III sample, as predicted, religious and spirituality struggle scores were more strongly correlated with SR subscale than TVR subscale scores. However, in each Stage III study, there were no differences between the association of TVR and SR scores with DAR-5 scores. This suggests that either TVR symptoms do not have separable construct validity, or the moral emotion of anger (and associated aggressive behaviors) is a shared element of SR and TVR outcomes from exposure to any type of PMIE among SMs and Veterans. Future research is needed to examine each of these possibilities and examine other unique convergent indicators of TVR scores (e.g., distrust, alienation, embitterment, grievance).

We demonstrated that PMIE endorsement was associated with substantially higher scores on the MIOS and greater functional impact relative to another type of stressor. *This validates the foundational assumption that MI is a PMIE-linked problem*. Generally, most participants had low or moderate scores. The is consistent with the hypothesis that clinically significant MI is a low-baserate problem (8). For epidemiological and clinical studies, future research will need to empirically test the predictive and clinical validity of variations in case definitions for MI, potentially using a combination of type of PMIE and threshold MIOS and functional impact scores, particularly as a means of distinguishing non-clinical levels of moral distress from MI (13). It is an empirical question whether requiring a PMIE to be a Criterion-A event or to occur in a life-threat context (or including positive endorsement of certain types of PTSD screener items) will improve the utility of a case definition. Perhaps more importantly, future research will need to test the incremental validity of MIOS scores, relative to PTSD symptoms indexed to a PMIE that meets or does not meet Criterion-A, as well as depression. In the US Stage III study, MIOS scores were particularly highly correlated with PTSD and depression, which suggests that when individuals are exposed to multiple types of PMIEs that occur in an enduring life-threat context the critical assessment task will be to determine if there is a worst and most currently distressing event that results in substantial impairment. Even when PTSD is the treatment focus, we predict that treatment will be impacted by impairing MI symptoms, which may require separate attention.

Although our study had unprecedented depth, it had limitations. First, although Stage I took 3 years to complete and entailed teams of clinical researchers in different countries, the qualitative results may have been different had an independent team of content experts examined the data and generated domain definitions. We also could have been more systematic about getting feedback about MIOS items and the MIOS from stakeholders. Second, although we used reputable survey firms who had established panels of SMs and Veterans, we cannot rule out the possibility that some responders may have provided different responses if interviews were conducted. Thankfully, research has shown that online responses do not substantively differ from paper and pencil and telephone-interview-based responses (54, 55). Also, for Stage III, we reduced the likelihood of fatigue and disengagement affecting test responses by randomly assigning test order. Finally, we found that participants had a sizable percentage of N/A entries for B-IPF scores, suggesting that, although there was a good deal of missing data in the Israeli and Australia studies, Stage III participants were not careless.

We anticipate that a wealth of research about the prevalence and predictors of MI will flourish using the MIOS and intervention studies will for the first time be able to track change using the MIOS. We also welcome clinicians using the MIOS to plan treatment and track clinical change over the course of treatment, which has been a missing link in any intervention approach that presumably targets MI. Yet, there are unaddressed empirical issues that arise from this study, some of which were described above. First, our results should be replicated with other samples, particularly among civilians and various occupational and non-English-speaking cultures. Second, research is needed to test the discriminant validity of the MIOS, which our group is planning to do. It will be important to examine the association between MIOS subscale scores and externalizing (including a cynical world view) and internalizing traits, given that it seems possible that externalizing would increase risk for exposure to TVR experiences and outcomes and internalizing would increase risk for exposure to SR experiences and outcomes. Finally, given that the studies described in this paper were all cross-sectional, future research should examine the causal direction between exposure to PMIEs and MIOS scores as well as the direction of the associations between converging indicators and MIOS scores.

## Data Availability Statement

The raw data supporting the conclusions of this article will be made available by the authors, once approved by the respective internal review boards.

## Ethics Statement

The studies involving human participants were reviewed and approved by VA Boston Healthcare System IRB, Australian Departments of Defense and Veterans' Affairs Human Research Ethics Committee, Western University HSREB, Royal Ottawa Research Ethics Board, Combat Stress Research Committee, and Ruppin Academic Center IRB. The patients/participants provided their written informed consent to participate in this study.

## Moral Injury Outcome Scale Consortium

Andrea Ashbaugh^1^, Julie Yeterian^2^, Danielle Berke^3^, Jessica Carney^4^, Mackenzie Cummings^5^, Ruth Chartoff^6^, Stephanie Ellickson-Larew^6, 7^, Breanna Grunthal^6^, Maya Bina N. Vannini^6^, Frank Weathers^8^, Luke Rusowicz-Orazem^6, 9^, Patrick Smith^10^, Fardous Hosseiny^10^, Lisa King^11^, J. Don Richardson^11−14^, Alexandra McIntyre-Smith, David Forbes^15, 16^, Kim Jones^15^, Ellie Lawrence-Wood^15^, Kelsey Madden^15^, Kim Murray^15^

^1^University of Ottawa, Ottawa, ON, Canada, ^2^William James College, Newton, MA, United States, ^3^Hunter College of the City University of New York, New York, NY, United States, ^4^University of Notre Dame, Notre Dame, IN, United States, ^5^Seattle Pacific University, Seattle, WA, United States, ^6^VA Boston Healthcare System, Boston, MA, United States, ^7^National Center for PTSD, Boston, MA, United States, ^8^VA Puget Sound Healthcare System, Seattle, WA, United States, ^9^School of Public Health, Boston University, Boston, MA, United States, ^10^Atlas Institute for Veterans and Families, Ottawa, ON, Canada, ^11^Operational Stress Injury Clinic, Parkwood Institute, London, ON, Canada, ^12^Franklin OSI Research Centre, Lawson Health Research Institute, London, ON, Canada, ^13^Department of Psychiatry, Western University, London, ON, Canada, ^14^Department of Psychiatry, McMaster University, Hamilton, ON, Canada, ^15^Phoenix Australia Centre for Posttraumatic Mental Health, Department of Psychiatry, Carlton, VA, Australia, ^16^University of Melbourne, Melbourne, VA, Australia

## Author Contributions

BL was the principal investigator, oversaw all aspects of data collection and analysis and principal writer of the manuscript. RP and AN analyzed data and co-wrote the manuscript. DM co-wrote the manuscript. AP helped secure funding and co-wrote the manuscript. AC analyzed the qualitative data and helped generate the MIOS and edited the manuscript. SH, LD, SF, GZ, and YL-B collected data, helped generate the scale, and co-wrote the manuscript. All authors contributed to the article and approved the submitted version.

## Funding

MIOS consortium activities were supported in part by VA Cooperative Studies Program, Office of Research and Development, US Department of Veterans Affairs; Department of Veterans' Affairs Australia, Phoenix Australia - Center for Posttraumatic Mental Health; and the Canadian Center of Excellence on PTSD and Related Mental Health Conditions.

## Conflict of Interest

The authors declare that the research was conducted in the absence of any commercial or financial relationships that could be construed as a potential conflict of interest.

## Publisher's Note

All claims expressed in this article are solely those of the authors and do not necessarily represent those of their affiliated organizations, or those of the publisher, the editors and the reviewers. Any product that may be evaluated in this article, or claim that may be made by its manufacturer, is not guaranteed or endorsed by the publisher.
