# Supplementary material for: Defining and Assessing the Syndrome of Moral Injury: Initial Findings of the Moral Injury Outcome Scale Consortium
Source: Front Psychiatry. 2022 Jul 5;13:923928. doi: 10.3389/fpsyt.2022.923928 (PMC9297368; doi:10.3389/fpsyt.2022.923928)
Supplement: Supplementary file 1 [file Data_Sheet_1.docx]

**Table S1**

*Breakdown of Consortium Sites’ Interview Contributions*

| Consortium Site | Interviewee Breakdown | | | |
| --- | --- | --- | --- | --- |
|  | Veterans | Active-Duty Service Members | Clinicians | Chaplains |
| United States |  |  |  |  |
| VA Boston Healthcare System | 4 | - | 6 | 10 |
| Minneapolis VA Healthcare System | - | - | 1 | - |
| San Francisco VA Healthcare System | - | - | 1 | - |
| VA San Diego Healthcare System | - | - | 1 | - |
| UTHESCA- Fort Hood Wyatt | 3 | 1 | 13 | - |
| Texas A&M University – San Antonio | 6 | - | - | - |
| Fargo VA Healthcare System | - | - | 7 | - |
| Canada |  |  |  |  |
| Operational Stress Injury (OSI) Clinic | - | - | 21 | - |
| Centre of Excellence on Post-Traumatic Stress Disorder | 11 | 6 | - | - |
| United Kingdom |  |  |  |  |
| Tyrwhitt House, Combat Stress | 6 | - | 4 | - |
| Australia |  |  |  |  |
| Phoenix Australia | 1 | - | 2 | - |
| Israel |  |  |  |  |
| Lior Tsfaty Center for Suicide and Mental Pain Studies & Ariel University | 20* | - | - | - |

*Note.* This first psychometric paper included English-speaking countries only.

| **Table S2**  *Domains of Impact and Subcomponents Within Domains* | | | | | | | | | | |  |  |
| --- | --- | --- | --- | --- | --- | --- | --- | --- | --- | --- | --- | --- |
| **Domains of Impact** | | | | | | | | | | |  |  |
| *Self-perception* | *Moral Thinking* | | | *Social Impacts* | *Self-harming / Self-sabotaging* | | | *Impairing Moral Emotions* | | *Beliefs about Meaning and Purpose* | |  |
| **Operational Definitions of Domains** | | | | | | | | | | |  |  |
| Alterations and disruptions in identity, which entail how individuals regard, understand, define, or see themselves (who they are), with respect to their core moral beliefs and what they are capable of | Changes in moral thinking, which entail the person judging situations or others moralistically and with condemnation | | | Alterations in degree of comfort with others, connectedness, social acceptance / belonging, changes in social behavior (e.g., the frequency and quality of engaging with others), trust in others and expectations of social safety | Deliberate and non-deliberate behaviors that negatively impact functioning, and impair health, personal safety, and quality of life / overall wellbeing | | | Predominant emotions and moods that arise when thinking of the event or that have been more prevalent since the event, as well as avoidance of emotions. Emotions/moods also include emotional behaviors and physiological reactions | | Alterations in individuals’ beliefs about life meaning or purpose, which may include religious or spiritual beliefs | |  |
| **Components** | | | | | | | | | | |  |  |
| Sense of self as bad | A hyper-focus about the morality of one’s own behavior | | | Lack of interest or investment in social relationships | Lack of self-care | | | Anger | | Loss of religious or spiritual beliefs or practices | | |
| Disruptions in global ideas about personal identity | Judgmental thoughts and appraisals of others | | | Loss of community | Disengaging from enjoyable activities | | | Shame | | Negatively changed relationships with God or divine forces | | |
|  |  | | |  |  | | |  | |  | | |
| **Table S2 - continued**  *Domains of Impact and Subcomponents of Moral Injury Outcome Scale* | | | | | | | | | | | | |
|  | | **Domains of Impact** | | | | | | |  | | | |
| *Self-perception* | | *Moral Thinking* | *Social Impacts* | | | *Self-harming / Self-sabotaging* | *Impairing Moral Emotions* | | *Beliefs about Meaning and Purpose* | | | |
| **Components** | | | | | | | | | | | | |
| Loss of trust in self as a moral agent | Constantly and deliberately thinking about the PMIE | | | Social isolation | Self-harm, para-suicidality, suicidal ideation, or suicidality | | | Disgust | | Loss of belief in the purpose or meaning of life | | |
| Feeling self-hate and unworthy | Since the event, repeatedly and deliberately thinking about morally challenging situations | | | Difficulty feeling or expressing emotions or empathy for others | Engaging in risky behaviors | | | Guilt | | Loss of belief that life is precious | | |
| Increased tendency to blame self | Expressions of reduced confidence in the value of morality and whether people can be good | | | Aggressive behavior toward others | Substance misuse | | | - | | - | | |
| - | - | | | Hiding/not disclosing significant feelings or events to others | Lack of purposeful behavior or motivation | | | - | | - | | |
| - | - | | | Expecting rejection or judgment | - | | | - | | - | | |
| - | - | | | Belief that people or institutions cannot be trusted to act morally | - | | | - | | - | | |

| **Table S3**  *Items Used in the Card Sort, Retained (or Altered), and Added to the 34-item MIOS, and the Preliminary 34-item MIOS* |
| --- |
| Items used in the Card Sort (Retained or Altered Items Bolded) |
| I am/feel unworthy of love and trust |
| I lost my sense of purpose **(I lost the feeling that I matter)** |
| Only bad people can do what I have done |
| **My actions don't fit with who I thought I was** |
| **I cannot accept myself** |
| I have not upheld my morals |
| I repeatedly go over what happened |
| I think about what I could have done differently **(I am preoccupied by how things should have gone differently)** |
| I do not know what it means to be good |
| **If people really knew me, they would not like me** |
| **I worry about what people think about me** |
| I am agitated. |
| **I feel guilty** |
| I blame myself |
| **I am/feel like a bad person** |
| **I am not the good person I thought I was** |
| I feel disconnected from myself |
| I lost my trust in my sense of right and wrong |
| I do not trust myself to make the right decisions |
| **I do not trust myself to be good** |
| I disgust myself |
| I cannot stop thinking about whether my actions are right or wrong |
| I cannot stop thinking about what happened |
| **I feel despair** |
| I cannot find meaning |
| When I see something wrong, I cannot stop thinking about it |
| **I am disconnected from other people** |
| **I cannot be honest with other people** |
| **I feel rejected by people** |
| **People would hate me if they really knew me.** |
| I do not trust authority figures |
| I am not obedient. |
| **I am disgusted by what happened** |
| **I feel guilty about what happened** |
| I regret constantly |
| **Table S3 - continued** |
| \| *Items Used in the Card Sort, Retained (or Altered), and Added to the 34-item MIOS, and the Preliminary 34-item MIOS* \| \| --- \| \| Items used in the Card Sort (Retained or Altered Items Bolded) \| |
| **I blame myself** |
| It is hard to live with myself |
| I am ashamed **(I am ashamed about what happened)** |
| **I am ashamed of my actions** |
| I try to hide my actions from others |
| There is no higher order to life **(I no longer believe there is a higher power)** |
| **I lost a sense of meaning in life** |
| **I am angry all of the time** |
| **I am quick to be angry** |
| **People need to be held accountable** |
| Wrongdoers should be punished **(People who break the rules should be punished)** |
| Authority figures cannot be trusted |
| I cannot trust others to do the morally right thing in difficult situations |
| I have lost faith in the goodness of humanity **(I have lost faith in humanity)** |
| Items Added to 34-item MIOS |
| **I keep myself from having success** |
| **I have trouble seeing goodness in others** |
| **People don’t deserve second chances** |
| **I feel like I don’t deserve a good life** |
| **I lost trust in others** |
| **I have lost the ability to forgive** |
| **I have lost pride in myself** |
| Preliminary 34-Item Moral Injury Outcome Scale Items and Their Domains of Impact |
| \| Item \| Domain of Impact \| \| --- \| --- \| \| 10. I feel like a bad person. \| Alterations in Self-Perception \| \| 16. I feel like I don’t deserve a good life. \| Alterations in Self-Perception \| \| 21. If people really knew me, they would not like me. \| Alterations in Self-Perception \| \| 28. I cannot accept myself. \| Alterations in Self-Perception \| \| 29. I am not the good person I thought I was. \| Alterations in Self-Perception \| \| 30. I do not trust myself to be good. \| Alterations in Self-Perception \| \| 32. I have lost pride in myself. \| Alterations in Self-Perception \| \| 33. My actions don't fit with who I thought I was. \| Alterations in Self-Perception \| \| 1. People need to be held accountable. \| Alterations in Moral Thinking \| \| 6. I have lost faith in humanity. \| Alterations in Moral Thinking \| \| 9. People who break the rules should be punished. \| Alterations in Moral Thinking \| \| 12. I have trouble seeing goodness in others. \| Alterations in Moral Thinking \| \| **Table S3 - continued**   \| *Items Used in the Card Sort, Retained (or Altered), and Added to the 34-item MIOS, and the Preliminary 34-item MIOS* \| \| --- \| \| \| \| Preliminary 34-Item Moral Injury Outcome Scale Items and Their Domains of Impact \| \| \| Item \| Domain of Impact \| \| 14. People don’t deserve second chances. \| Alterations in Moral Thinking \| \| 22. I lost trust in others. \| Alterations in Moral Thinking \| \| 23. I have lost the ability to forgive. \| Alterations in Moral Thinking \| \| 8. People would hate me if they really knew me. \| Social Impacts \| \| 11. I am disconnected from other people. \| Social Impacts \| \| 17. I worry about what people think about me. \| Social Impacts \| \| 24. I feel rejected by people. \| Social Impacts \| \| 34. I cannot be honest with other people. \| Social Impacts \| \| 2. I am preoccupied by how things should have gone differently. \| Self-harming and Self-sabotaging Behaviors \| \| 18. I keep myself from having success. \| Self-harming and Self-sabotaging Behaviors \| \| 3. I blame myself. \| Emotional Aftermath \| \| 4. I feel guilty about what happened. \| Emotional Aftermath \| \| 5. I am angry all the time. \| Emotional Aftermath \| \| 7. I feel despair. \| Emotional Aftermath \| \| 13. I am ashamed about what happened. \| Emotional Aftermath \| \| 15. I am disgusted by what happened. \| Emotional Aftermath \| \| 19. I am ashamed of my actions. \| Emotional Aftermath \| \| 25. I feel guilty. \| Emotional Aftermath \| \| 27. I am quick to be angry. \| Emotional Aftermath \| \| 20. I no longer believe there is a higher power. \| Beliefs about Life Meaning and Purpose \| \| 26. I lost the feeling that I matter. \| Beliefs about Life Meaning and Purpose \| \| 31. I lost a sense of meaning in life. \| Beliefs about Life Meaning and Purpose \|   *Note.* Item numbers represent order in which they were presented to participants. |

**Scale S1**

**Moral Injury Outcome Scale***

**Instructions: This questionnaire asks about experiences you may have had after a very stressful experience in which:**

- **You did something (or failed to do something) that went against your moral code or values (e.g., you harmed someone or failed to protect someone from harm), or**
- **You saw someone (or people) do something or fail to do something that went against your moral code or values (e.g., you witnessed cruel behavior), or**
- **You were directly affected by someone doing something or failing to do something that went against your moral code or values (e.g., being betrayed by someone you trusted).**

**Have you had an experience (or experiences) as described above?**

| **____Yes**  **Please answer questions A-C while thinking about the worst event that currently bothers you the most. This could be one of the examples above, or some other very stressful experience that went against your core values.**  ***A. Did the event involve something you did or failed to do?***  **_____Yes _____No**  ***B. Did the event involve observing someone else acting (or failing to act)?***  **_____Yes _____No**  ***C. Did the event involve being directly impacted by someone else (or people) acting (or failing to act)?***  **_____Yes _____No**  ***For events that had multiple features, which aspect was the worst (A, B, or C)* _____*?***  **Please also answer questions 1-3 below **:** | **____No**  **If you experienced other types of very stressful events, please answer questions 1-3 below about the worst and most currently distressing event (and continue to the next page).** |
| --- | --- |
| **1. *What year did this event happen __________?***  **2*. Did the event involve actual or threatened death, serious injury, or sexual violence?***  **_____Yes _____No**  **3. *In the past month, have you…***   1. **had nightmares about the event or thought about the event when you did not want to?**   **_____Yes _____No**   1. **tried hard not to think about the event or went out of your way to avoid situations that reminded you of the event(s)?**   **_____Yes _____No**   1. **been constantly on guard, watchful, or easily startled?**   **_____Yes _____No**   1. **felt numb or detached from people, activities, or your surroundings?**   **_____Yes _____No**   1. **felt guilty or unable to stop blaming yourself or others for the event(s) or any problems the event(s) may have caused?**   **_____Yes _____No**  **PLEASE GO TO NEXT PAGE**  ***If you feel comfortable, please briefly describe the worst event referenced on the first page:*** | |

**Keeping this worst event in mind, please read each of these statements and circle one of the numbers to the right to indicate how much you would agree with the statement in the past month.**

| ***In the past month, how strongly would you agree with the following statements:*** | ***Strongly Disagree*** | ***Disagree*** | ***Neither Agree or Disagree*** | ***Agree*** | ***Strongly Agree*** |
| --- | --- | --- | --- | --- | --- |
| 1. **I blame myself.** | **0** | **1** | **2** | **3** | **4** |
| 1. **I have lost faith in humanity.** | **0** | **1** | **2** | **3** | **4** |
| 1. **People would hate me if they really knew me.** | **0** | **1** | **2** | **3** | **4** |
| 1. **I have trouble seeing goodness in others.** | **0** | **1** | **2** | **3** | **4** |
| 1. **People don’t deserve second chances.** | **0** | **1** | **2** | **3** | **4** |
| 1. **I am disgusted by what happened.** | **0** | **1** | **2** | **3** | **4** |
| 1. **I feel like I don’t deserve a good life.** | **0** | **1** | **2** | **3** | **4** |
| 1. **I keep myself from having success.** | **0** | **1** | **2** | **3** | **4** |
| 1. **There is no higher power.** | **0** | **1** | **2** | **3** | **4** |
| 1. **I lost trust in others.** | **0** | **1** | **2** | **3** | **4** |
| 1. **I am angry all the time.** | **0** | **1** | **2** | **3** | **4** |
| 1. **I am not the good person I thought I was.** | **0** | **1** | **2** | **3** | **4** |
| 1. **I have lost pride in myself.** | **0** | **1** | **2** | **3** | **4** |
| 1. **I cannot be honest with other people.** | **0** | **1** | **2** | **3** | **4** |

**Brief Inventory of Psychosocial Functioning*****

**Please write in a number for each item below that represents how much these experiences have made it hard for you to function in each of the following areas (if not applicable, use N/A):**

**Not at All               Somewhat            Extremely**

**0         1         2         3        4        5          6**

1. **Romantic relationships with spouse or partner _____**
2. **Relationships with your children _____**
3. **Family relationships _____**
4. **Friendships or socializing _____**
5. **Work _____**
6. **Training or education _____**
7. **Day to day activities, such as chores, errands, managing medical care _____**

**The Moral Injury Outcome Scale* (2021). Litz, B.T., Phelps, A., Frankfurt, S., Murphy, D. Nazarov, A. Houle, S., Levi-Belz, Y., Zerach, G., Dell, L., Hosseiny, F., and the members of the *Moral Injury Outcome Scale (MIOS) Consortium.* MIOS consortium activities were supported in part by VA Cooperative Studies Program, Office of Research and Development, US Department of Veterans Affairs; Veterans Affairs Australia, Phoenix Centre for Posttraumatic Mental Health; and the Canadian Centre of Excellence on PTSD and Related Mental Health Conditions. ** Prins et al. (2016) *** Kleiman et al. (2020).

**Figure S1**

*Moral Injury Outcome Scale Confirmatory Factor Analysis for Stage II: United States, United Kingdom , and Ottawa*


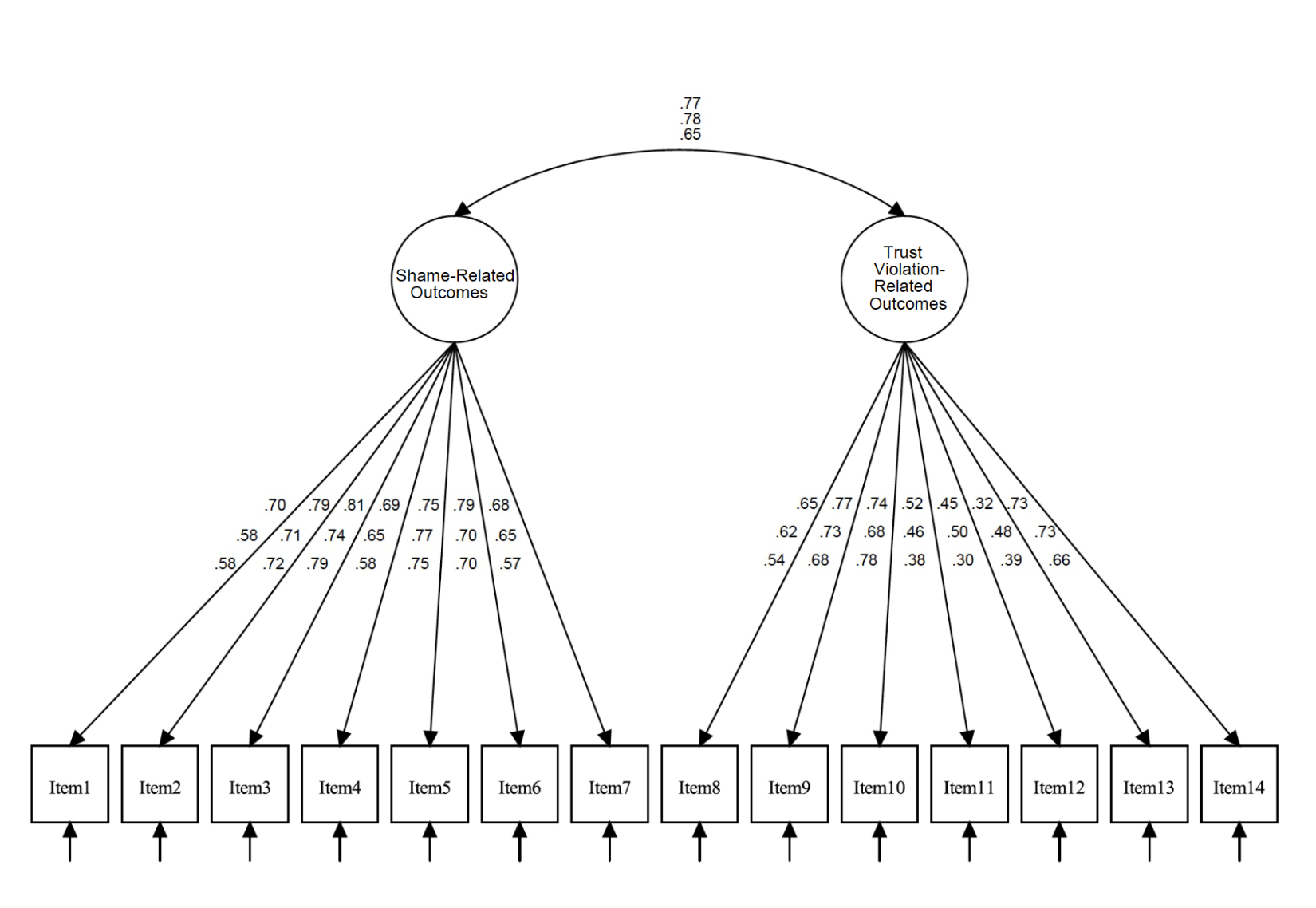


*Note.* Top row of factor loadings represents USA; middle row represents the UK; bottom row represents Ottawa. Factor loadings are standardized. All loadings significant at *p* < .001.

**Table S4**

*Pattern Matrix Factor Loadings for Preliminary 34-Item Moral Injury Outcome Scale*

| Item | Shame-related Outcomes | Trust Violation-related  Outcomes |
| --- | --- | --- |
| I am ashamed of my actions. | **.89** | -.20 |
| My actions don't fit with who I thought I was. | **.86** | -.09 |
| I am not the good person I thought I was. | **.83** | -.03 |
| I feel guilty. | **.81** | -.04 |
| I feel like a bad person. | **.79** | .02 |
| I do not trust myself to be good. | **.79** | -.05 |
| I cannot accept myself. | **.76** | .13 |
| I am ashamed about what happened. | **.74** | -.05 |
| If people really knew me, they would not like me. 8. People would hate me if they really knew me. | **.72** | .06 |
| People would hate me if they really knew me. | **.71** | .10 |
| I feel like I don’t deserve a good life. | **.70** | .08 |
| I blame myself. | **.69** | -.04 |
| I feel guilty about what happened. | **.68** | -.06 |
| I have lost pride in myself. | **.68** | .21 |
| I keep myself from having success. | **.67** | .12 |
| I lost the feeling that I matter. | **.56** | **.39** |
| I lost a sense of meaning in life. | **.55** | **.38** |
| I cannot be honest with other people. | **.54** | .11 |
| I feel rejected by people. | **.54** | **.35** |
| I worry about what people think about me. | **.47** | .12 |
| I lost trust in others. | .20 | **.68** |
| I have lost faith in humanity. | .16 | **.68** |
| I have lost the ability to forgive. | .23 | **.65** |
| I have trouble seeing goodness in others. | .21 | **.63** |
| I am angry all the time. | **.33** | **.56** |
| I feel despair. | **.39** | **.55** |
| I am disconnected from other people. | **.36** | **.54** |
| I am quick to be angry. | **.35** | **.46** |
| I am disgusted by what happened. | .21 | **.39** |
| People who break the rules should be punished. | -.05 | **.38** |
| People need to be held accountable. | -.20 | **.36** |
| I am preoccupied by how things should have gone differently. | **.34** | **.34** |
| People don’t deserve second chances. | .18 | **.30** |
| I no longer believe there is a higher power. | .16 | .21 |

*Note.* Bolded values represent loadings ≥ .30.

***Table S5****Moral Injury Outcome Scale Descriptive Statistics and Inter-Item/Item-Total Correlations: Stage II – United Kingdom*

| Item | *M (SD)* | 1 | 2 | 3 | 4 | 5 | 6 | 7 | 8 | 9 | 10 | 11 | 12 | 13 | 14 |
| --- | --- | --- | --- | --- | --- | --- | --- | --- | --- | --- | --- | --- | --- | --- | --- |
| 1. I blame myself. | 2.54 (1.24) | 1 |  |  |  |  |  |  |  |  |  |  |  |  |  |
| 2. People would hate me if they really knew me. | 2.36 (1.24) | .43 | 1 |  |  |  |  |  |  |  |  |  |  |  |  |
| 3. I feel like I don’t deserve a good life. | 2.14 (1.31) | .50 | .30 | 1 |  |  |  |  |  |  |  |  |  |  |  |
| 4. I keep myself from having success. | 2.44 (1.08) | .42 | .42 | .53 | 1 |  |  |  |  |  |  |  |  |  |  |
| 5. I am not the good person I thought I was. | 2.25 (1.20) | .46 | .58 | .60 | .46 | 1 |  |  |  |  |  |  |  |  |  |
| 6. I have lost pride in myself. | 2.61 (1.19) | .39 | .39 | .53 | .53 | .58 | 1 |  |  |  |  |  |  |  |  |
| 7. I cannot be honest with other people. | 1.95 (1.22) | .29 | .48 | .43 | .43 | .57 | .41 | 1 |  |  |  |  |  |  |  |
| 8. I am angry all the time. | 2.23 (1.14) | .20 | .40 | .32 | .34 | .36 | .36 | .32 | 1 |  |  |  |  |  |  |
| 9. I have lost faith in humanity. | 2.62 (1.13) | .20 | .48 | .36 | .36 | .32 | .38 | .28 | .50 | 1 |  |  |  |  |  |
| 10. I have trouble seeing goodness in others. | 2.34 (1.14) | .22 | .35 | .43 | .24 | .34 | .36 | .39 | .39 | .51 | 1 |  |  |  |  |
| 11. People don’t deserve second chances. | 1.77 (1.14) | .16 | .21 | .40 | .24 | .28 | .27 | .24 | .27 | .30 | .34 | 1 |  |  |  |
| 12. I am disgusted by what happened. | 2.68 (1.11) | .35 | .31 | .38 | .34 | .31 | .35 | .29 | .28 | .33 | .29 | .36 | 1 |  |  |
| 13. There is no higher power. | 2.17 (1.32) | .18 | .29 | .30 | .35 | .32 | .32 | .38 | .26 | .33 | .33 | .23 | .20 | 1 |  |
| 14. I lost trust in others. | 2.80 (1.05) | .21 | .45 | .40 | .38 | .42 | .42 | .38 | .44 | .56 | .54 | .28 | .30 | .34 | 1 |
| 15. Total MIOS | 32.87 (10.54) | .53 (.57) | .68 (.71) | .72 (.75) | .63  (.66) | .72 (.74) | .66 (.69) | .63 (.66) | .55 (.58) | .62  (.65) | .60 (.64) | .52 (.56) | .52 (.56) | .50 (.54) | .65  (.67) |

*Note*. All correlations significant at *p* < .001. Uncorrected item-total correlations in brackets (else are corrected item-total correlations).

***Table S6***

*Moral Injury Outcome Scale Descriptive Statistics and Inter-Item/Item-Total Correlations: Stage II – United States of America*

| Item | *M (SD)* | 1 | 2 | 3 | 4 | 5 | 6 | 7 | 8 | 9 | 10 | 11 | 12 | 13 | 14 |
| --- | --- | --- | --- | --- | --- | --- | --- | --- | --- | --- | --- | --- | --- | --- | --- |
| 1. I blame myself. | 1.80 (1.33) | 1 |  |  |  |  |  |  |  |  |  |  |  |  |  |
| 2. People would hate me if they really knew me. | 1.52 (1.18) | .54 | 1 |  |  |  |  |  |  |  |  |  |  |  |  |
| 3. I feel like I don’t deserve a good life. | 1.39 (1.19) | .57 | .66 | 1 |  |  |  |  |  |  |  |  |  |  |  |
| 4. I keep myself from having success. | 1.71 (1.23) | .52 | .49 | .58 | 1 |  |  |  |  |  |  |  |  |  |  |
| 5. I am not the good person I thought I was. | 1.58 (1.23) | .57 | .62 | .62 | .46 | 1 |  |  |  |  |  |  |  |  |  |
| 6. I have lost pride in myself. | 1.79 (1.27) | .55 | .58 | .63 | .57 | .61 | 1 |  |  |  |  |  |  |  |  |
| 7. I cannot be honest with other people. | 1.53 (1.25) | .47 | .59 | .52 | .50 | .50 | .49 | 1 |  |  |  |  |  |  |  |
| 8. I am angry all the time. | 1.72 (1.17) | .32 | .46 | .41 | .37 | .39 | .45 | .43 | 1 |  |  |  |  |  |  |
| 9. I have lost faith in humanity. | 2.15 (1.25) | .31 | .45 | .47 | .38 | .38 | .53 | .40 | .45 | 1 |  |  |  |  |  |
| 10. I have trouble seeing goodness in others. | 1.96 (1.22) | .31 | .44 | .48 | .46 | .36 | .45 | .44 | .52 | .58 | 1 |  |  |  |  |
| 11. People don’t deserve second chances. | 1.18 (1.00) | .28 | .38 | .39 | .30 | .28 | .34 | .35 | .35 | .38 | .38 | 1 |  |  |  |
| 12. I am disgusted by what happened. | 2.68 (1.11) | .38 | .23 | .28 | .26 | .26 | .38 | .26 | .29 | .32 | .27 | .20 | 1 |  |  |
| 13. There is no higher power. | 2.17 (1.30) | .17 | .23 | .21 | .14 | .24 | .29 | .25 | .18 | .28 | .18 | .16 | .15 | 1 |  |
| 14. I lost trust in others. | 2.80 (1.05) | .34 | .38 | .39 | .41 | .33 | .46 | .40 | .46 | .61 | .53 | .37 | .40 | .21 | 1 |
| 15. Total MIOS | 25.14 (11.36) | .65  (.69) | .73  (.76) | .75  (.77) | .66  (.69) | .69  (.71) | .78  (.79) | .68  (.71) | .62  (.65) | .67  (.70) | .66  (.69) | .51  (.54) | .46  (.50) | .36  (.41) | .64  (.67) |

*Note*. All correlations significant at *p* < .001. Uncorrected item-total correlations in brackets (else are corrected item-total correlations).

***Table S7***

| Item | *M (SD)* | 1 | 2 | 3 | 4 | 5 | 6 | 7 | 8 | 9 | 10 | 11 | 12 | 13 | 14 |
| --- | --- | --- | --- | --- | --- | --- | --- | --- | --- | --- | --- | --- | --- | --- | --- |
| 1. I blame myself. | 2.04 (1.22) | 1 |  |  |  |  |  |  |  |  |  |  |  |  |  |
| 2. People would hate me if they really knew me. | 1.49 (1.12) | .40*** | 1 |  |  |  |  |  |  |  |  |  |  |  |  |
| 3. I feel like I don’t deserve a good life. | 1.26 (1.01) | .47*** | .57*** | 1 |  |  |  |  |  |  |  |  |  |  |  |
| 4. I keep myself from having success. | 1.90 (1.17) | .32*** | .41*** | .41*** | 1 |  |  |  |  |  |  |  |  |  |  |
| 5. I am not the good person I thought I was. | 1.69 (1.14) | .38*** | .55*** | .61*** | .43*** | 1 |  |  |  |  |  |  |  |  |  |
| 6. I have lost pride in myself. | 2.10 (1.17) | .42*** | .46*** | .51*** | .51*** | .56*** | 1 |  |  |  |  |  |  |  |  |
| 7. I cannot be honest with other people. | 1.50 (1.12) | .37*** | .44*** | .49*** | .32*** | .40*** | .34*** | 1 |  |  |  |  |  |  |  |
| 8. I am angry all the time. | 2.10 (1.09) | .35*** | .32*** | .29*** | .24*** | .26*** | .30*** | .23*** | 1 |  |  |  |  |  |  |
| 9. I have lost faith in humanity. | 2.29 (1.14) | .32*** | .25*** | .32*** | .13* | .27*** | .23*** | .21** | .40*** | 1 |  |  |  |  |  |
| 10. I have trouble seeing goodness in others. | 2.17 (1.20) | .34*** | .37*** | .42*** | .25*** | .38*** | .37*** | .29*** | .39*** | .53** | 1 |  |  |  |  |
| 11. People don’t deserve second chances. | 1.25 (0.88) | .16* | .19** | .29*** | .03 | .19** | .14* | .21** | .17** | .23*** | .32*** | 1 |  |  |  |
| 12. I am disgusted by what happened. | 2.90 (1.21) | .10 | .06 | .15* | .14* | .08 | .12 | -.03 | .03 | .24*** | .20** | .18** | 1 |  |  |
| 13. There is no higher power. | 1.89 (1.30) | .03 | .15* | .28*** | .11 | .17** | .17** | .16* | .28*** | .29*** | .30*** | .26*** | -.09 | 1 |  |
| 14. I lost trust in others. | 2.73 (1.04) | .27*** | .33*** | .29*** | .31*** | .25*** | .40*** | .24*** | .34*** | .46*** | .51*** | .17** | .34*** | .20** | 1 |
| 15. Total MIOS | 27.32 (9.08) | .57  (.61)*** | .64 (.68)*** | .72  (.74)*** | .53 (.57)*** | .65  (.68)*** | .65 (.69)*** | .53  (.57)*** | .52 (.57)*** | .56  (.60)*** | .66 (.69)*** | .25  (.30)*** | .36 (.42)*** | .59  (.62)*** | 1 |

*Moral Injury Outcome Scale Descriptive Statistics and Inter-Item/Item-Total Correlations: Stage II – Ottawa*

*Note. ***p*<.001, ***p<*.01, **p<*.05.

***Table S8***

| Item | *M (SD)* | 1 | 2 | 3 | 4 | 5 | 6 | 7 | 8 | 9 | 10 | 11 | 12 | 13 | 14 |
| --- | --- | --- | --- | --- | --- | --- | --- | --- | --- | --- | --- | --- | --- | --- | --- |
| 1. I blame myself. | 1.97 (1.30) | 1 |  |  |  |  |  |  |  |  |  |  |  |  |  |
| 2. People would hate me if they really knew me. | 1.49 (1.19) | .38*** | 1 |  |  |  |  |  |  |  |  |  |  |  |  |
| 3. I feel like I don’t deserve a good life. | 1.29 (1.20) | .39*** | .52*** | 1 |  |  |  |  |  |  |  |  |  |  |  |
| 4. I keep myself from having success. | 2.10 (1.22) | .35*** | .36*** | .43*** | 1 |  |  |  |  |  |  |  |  |  |  |
| 5. I am not the good person I thought I was. | 1.67 (1.18) | .49*** | .68*** | .59*** | .40*** | 1 |  |  |  |  |  |  |  |  |  |
| 6. I have lost pride in myself. | 2.23 (1.24) | .41*** | .47*** | .47*** | .45*** | .66*** | 1 |  |  |  |  |  |  |  |  |
| 7. I cannot be honest with other people. | 1.72 (1.18) | .33*** | .39*** | .35*** | .29** | .49*** | .46*** | 1 |  |  |  |  |  |  |  |
| 8. I am angry all the time. | 2.19 (1.07) | .27** | .19* | .24** | .38*** | .28** | .39*** | .29** | 1 |  |  |  |  |  |  |
| 9. I have lost faith in humanity. | 2.20 (1.15) | .27** | .21* | .34*** | .41*** | .35*** | .47*** | .24** | .56*** | 1 |  |  |  |  |  |
| 10. I have trouble seeing goodness in others. | 2.15 (1.20) | .19* | .22* | .31*** | .39*** | .28** | .39*** | .39*** | .53*** | .68*** | 1 |  |  |  |  |
| 11. People don’t deserve second chances. | 1.35 (1.00) | .18 | .25** | .32*** | .36*** | .38*** | .28** | .25** | .40*** | .39*** | .37*** | 1 |  |  |  |
| 12. I am disgusted by what happened. | 3.00 (1.09) | .24* | .07 | .09 | .29** | .07 | .34*** | .20* | .33*** | .40*** | .28** | .33*** | 1 |  |  |
| 13. There is no higher power. | 2.10 (1.35) | .06 | .06 | -.002 | .17 | .20* | .04 | .15 | .28** | .24* | .18 | .37*** | .18 | 1 |  |
| 14. I lost trust in others. | 2.81 (1.01) | .15 | .13 | .22* | .42*** | .17 | .41*** | .26** | .42*** | .59*** | .56*** | .26** | .40*** | .23* | 1 |
| 15. Total MIOS | 28.27 (9.98) | .52  (.56)*** | .54  (.58)*** | .58  (.62)*** | .63  (.67)*** | .68  (.72)*** | .70  (.73)*** | .56  (.60)*** | .61  (.64)*** | .68  .(71)*** | .63  (.67)*** | .56  (.59)*** | .44  (.48)*** | .31  (.38)*** | .56  (.60)*** |

*Moral Injury Outcome Scale Descriptive Statistics and Inter-Item/Item-Total Correlations: Stage II – Australia*

*Note. ***p*<.001, ***p<*.01, **p<*.05

**Table S9**

*Stage III Sociodemographic Characteristics of US PMIE Endorsing Participants vs. Non-PMIE Endorsing Participants*

| Demographic Characteristic | PMIE Endorsers  (*N* = 350) | | Non-PMIE Endorsers  (*N* = 70) | | Difference | Chi-squared | Significance level |
| --- | --- | --- | --- | --- | --- | --- | --- |
|  | *n* | % | *n* | % | % | *χ2* | *p* |
| Veteran | 264 | 75.4 | 53 | 75.7 | 0.3 | 0.003 | .96 |
| Active Duty Service Member | 86 | 24.6 | 17 | 24.3 | 0.3 | 0.003 | .96 |
| Gender |  |  |  |  |  |  |  |
| Female | 57 | 16.3 | 10 | 14.3 | 2.0 | 0.174 | .68 |
| Male | 291 | 83.1 | 59 | 84.3 | 1.2 | 0.055 | .81 |
| Transgender Man | 0 | 0 | 1 | 1.4 | 1.4 | 5.012 | .03* |
| Transgender Woman | 1 | 0.3 | 0 | 0 | 0.3 | 0.200 | .65 |
| Non-binary | 1 | 0.3 | 0 | 0 | 0.3 | 0.200 | .65 |
| Age Range |  |  |  |  |  |  |  |
| 18-19 | 2 | 0.6 | 0 | 0 | 0.6 | 0.402 | .53 |
| 20-29 | 20 | 5.7 | 4 | 5.7 | 0 | - | - |
| 30-39 | 148 | 42.3 | 24 | 34.3 | 8.0 | 1.544 | .21 |
| 40-49 | 144 | 41.1 | 24 | 34.3 | 6.7 | 1.143 | .29 |
| 50-59 | 32 | 9.1 | 7 | 10 | 0.9 | 0.051 | .82 |
| 60-69 | 4 | 1.1 | 7 | 10 | 8.9 | 17.94 | <.01* |
| 70-79 | 0 | 0 | 4 | 5.7 | 5.7 | 20.19 | <.01* |
| Race |  |  |  |  |  |  |  |
| American Indian/ Alaska Native | 3 | 0.9 | 1 | 1.4 | 0.5 | 0.202 | .65 |
| Black/African American | 33 | 9.4 | 5 | 7.1 | 2.3 | 0.370 | .54 |
| Asian | 6 | 1.7 | 0 | 0 | 1.7 | 1.217 | .27 |
| Hispanic/Latino | 25 | 7.1 | 0 | 0 | 7.1 | 5.317 | .02* |
| Native Hawaiian/ Other Pacific Islander | 1 | 0.3 | 0 | 0 | 0.3 | 0.200 | .65 |
| White | 265 | 75.7 | 64 | 91.4 | 15.7 | 8.487 | <.01* |
| Mixed Race | 15 | 4.3 | 0 | 0 | 4.3 | 3.111 | .08 |
| Other | 2 | 0.6 | 0 | 0 | 0.6 | 0.402 | .53 |
| Years Deployed |  |  |  |  |  |  |  |
| 2001-2010 | 214 | 61.1 | 42 | 60 | 1.1 | 0.018 | .89 |
| 2011-2021 | 136 | 38.9 | 27 | 38.6 | 0.3 | 0.001 | .98 |
| Times Deployed |  |  |  |  |  |  |  |
| 1-5 | 267 | 76.3 | 58 | 82.9 | 6.6 | 1.186 | .28 |
| 6-10 | 42 | 12 | 6 | 0.9 | 11.1 | 0.668 | .41 |
| 11+ | 41 | 11.7 | 6 | 0.9 | 10.8 | 0.646 | .42 |
| Combat Arms | 271 | 77.4 | 40 | 57.1 | 20.3 | 7.593 | .01* |

*Note.* PMIE = Potentially Morally Injurious Event.

*Statistically significant at the 95% confidence level.

**Table S10**

*Stage III Sociodemographic Characteristics of Israeli PMIE Endorsing Participants vs. Non-PMIE Endorsing Participants*

| Demographic Characteristic | PMIE Endorsers  (*N* = 71) | | Non-PMIE Endorsers  (*N* = 40) | | Difference | Chi-squared | Significance level |
| --- | --- | --- | --- | --- | --- | --- | --- |
|  | *n* | % | *n* | % | % | *χ2* | *p* |
| Military Status |  |  |  |  |  |  |  |
| IDF Veteran | 65 | 91.5 | 39 | 97.5 | 6.0 | 1.533 | .22 |
| IDF Active Service  Member | 6 | 8.5 | 1 | 2.5 | 6.0 | 1.533 | .22 |
| Gender |  |  |  |  |  |  |  |
| Female | 15 | 21.1 | 4 | 10.0 | 11.1 | 2.233 | .14 |
| Male | 53 | 74.6 | 36 | 90.0 | 15.4 | 3.795 | .05 |
| Prefer not to answer | 3 | 4.2 | 0 | 0.0 | 4.2 | 1.737 | .19 |
| Age Range |  |  |  |  |  |  |  |
| 18-19 | 0 | 0 | 0 | 0.0 | 0.0 | 0 | 1.0 |
| 20-29 | 64 | 90.1 | 40 | 100.0 | 9.9 | 4.209 | .04* |
| 30-39 | 6 | 8.5 | 0 | 0.0 | 8.5 | 3.573 | .06 |
| 40-49 | 1 | 1.4 | 0 | 0.0 | 1.4 | 0.569 | .45 |
| 50-59 | 0 | 0 | 0 | 0.0 | 0.0 | 0.0 | 1.0 |
| 60-69 | 0 | 0 | 0 | 0.0 | 0.0 | 0.0 | 1.0 |
| 70-79 | 0 | 0 | 0 | 0.0 | 0.0 | 0.0 | 1.0 |
| Race |  |  |  |  |  |  |  |
| Israeli | 66 | 93.0 | 35 | 87.5 | 5.5 | 0.930 | .34 |
| American | 2 | 2.8 | 2 | 5.5 | 2.7 | 0.351 | .55 |
| African | 2 | 2.8 | 0 | 0.0 | 2.8 | 1.147 | .28 |
| European | 1 | 1.4 | 3 | 7.5 | 6.1 | 2.733 | .10 |
| Served in the IDF over the last 20 years | 71 | 100.0 | 40 | 100.0 | 0.0 | 0.0 | 1.0 |

*Note.* PMIE = Potentially Morally Injurious Event. IDF = Israeli Defense Force

*Statistically significant at the 95% confidence level.

**Table S11**

*Stage III Sociodemographic Characteristics of Australian PMIE Endorsing Participants vs. Non-PMIE Endorsing Participants.*

| Demographic Characteristic | PMIE Endorsers (*N*=91) | | PMIE Non-Endorsers (*N*=13) | | Difference | Chi-squared | Significance Level |
| --- | --- | --- | --- | --- | --- | --- | --- |
|  | *n* | % | *n* | % | % | *χ2* | *p* |
| Military Status |  |  |  |  |  |  |  |
| Veteran Defence member | 57 | 62.6 | 8 | 61.5 | 1.1 | 0 | 1.0 |
| Active Defence member | 34 | 37.4 | 5 | 38.5 | 1.1 | 0 | 1.0 |
| Gender |  |  |  |  |  |  |  |
| Female | 24 | 26.4 | 1 | 7.7 | 18.7 | 1.27 | .26 |
| Male | 67 | 73.6 | 12 | 92.3 | 18.7 | 1.27 | .26 |
| Age Range |  |  |  |  |  |  |  |
| 18-19 | 0 | 0 | 1 | 7.7 | 7.7 | 1.30 | .26 |
| 20-29 | 0 | 0 | 0 | 0 | 0 | - | - |
| 30-39 | 20 | 22.0 | 1 | 7.7 | 14.3 | .69 | .41 |
| 40-49 | 27 | 29.7 | 1 | 7.7 | 22 | 1.79 | .18 |
| 50-59 | 27 | 29.7 | 6 | 46.2 | 16.5 | .77 | .38 |
| 60-69 | 12 | 13.2 | 0 | 0 | 13.2 | .86 | .35 |
| 70-79 | 4 | 4.4 | 4 | 30.8 | 26.4 | 7.74 | .005 |
| 80+ | 1 | 1.1 | 0 | 0 | 1.1 | 0 | 1.0 |
| Branch |  |  |  |  |  |  |  |
| Navy | 27 | 29.7 | 7 | 53.8 | 24.1 | 2.02 | .16 |
| Army | 48 | 52.7 | 5 | 38.5 | 14.2 | .45 | .51 |
| Air Force | 16 | 17.6 | 1 | 7.7 | 9.9 | .25 | .62 |
| Rank |  |  |  |  |  |  |  |
| General Enlistee | 20 | 22.0 | 3 | 23.1 | 1.1 | 0 | 1.0 |
| Commissioned Officer | 34 | 37.4 | 7 | 53.8 | 16.4 | .70 | .40 |
| Non-Commissioned Officer | 37 | 40.7 | 3 | 23.1 | 17.6 | .84 | .36 |
| Years Served in the ADF |  |  |  |  |  |  |  |
| 0-5 years | 13 | 14.3 | 4 | 30.8 | 16.5 | 1.22 | .27 |
| 6-10 years | 12 | 13.2 | 1 | 7.7 | 5.5 | .01 | .91 |
| 11-20 years | 27 | 29.7 | 4 | 30.8 | 1.1 | 0 | 1.0 |
| 20+ years | 39 | 42.9 | 4 | 30.8 | 12.1 | .28 | .60 |
| Deployment History |  |  |  |  |  |  |  |
| Never Been Deployed | 23 | 25.3 | 2 | 15.4 | 9.9 | .19 | .67 |
| Have Been Deployed | 68 | 74.7 | 11 | 84.6 | 9.9 | .19 | .67 |
| For War-like Operations | 49 | 72.1 | 9 | 81.8 | 9.7 | .10 | .76 |
| For Peacekeeping Operations | 40 | 58.8 | 2 | 18.2 | 40.6 | 4.76 | .03 |
| For Humanitarian Operations | 18 | 26.5 | 1 | 9.1 | 17.4 | .76 | .38 |
| For Border Protection | 15 | 22.1 | 1 | 9.1 | 13 | .35 | .56 |
| Rather Not Specify | 5 | 7.4 | 2 | 18.2 | 10.8 | .36 | .55 |
| Number of Deployments^a^ |  |  |  |  |  |  |  |
| 1-5 | 44 | 64.7 | 10 | 90.9 | 26.2 | 1.92 | .17 |
| 6-10 | 11 | 16.2 | 1 | 9.1 | 7.1 | .02 | .88 |
| 11+ | 1 | .01 | 0 | 0 | .01 | 0 | 1.0 |
| Did not respond | 12 | 17.6 | 0 | 0 | 17.6 | 1.12 | .29 |

*Note.* ADF = Australian Defence Forces. PMIE=Potentially Morally Injurious Event.

^a^Values reported below are for participants who have been deployed (*n*=68 for PMIE Endorsers; *n*=11 for PMIE Non-Endorsers).

**Figure S2**

*Moral Injury Outcome Scale Confirmatory Factor Analysis: Phase III United States*

**
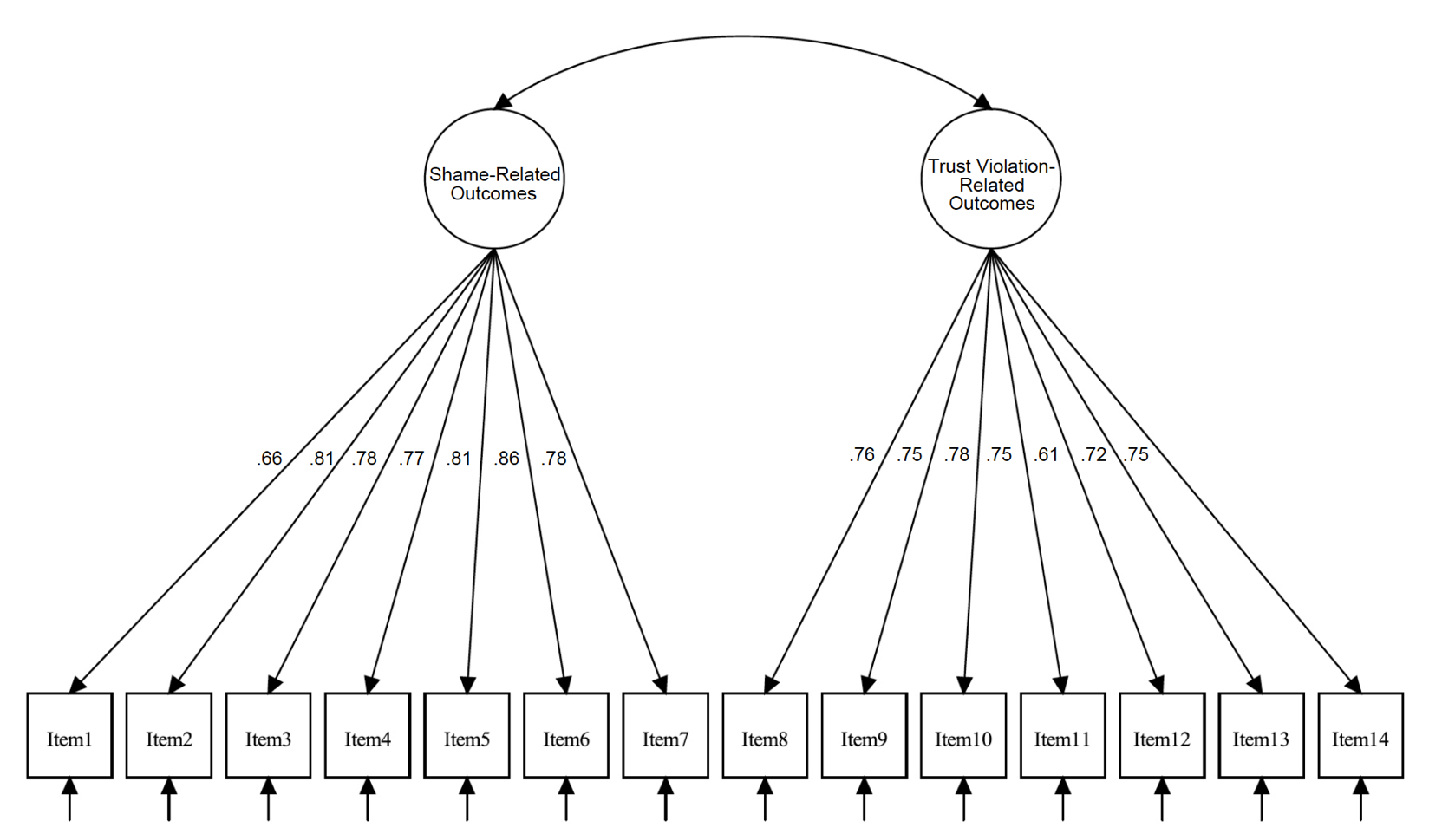
**

*Note.* Fit indices were as follows: χ2(76) = 376.99, p < .001, RMSEA = .097 (90% CI = .087, .107), CFI = .925, TLI = .910. All loadings significant at *p* < .001.

|  | PMIE Endorsers  (*N* = 350) | | | | |  | PMIE Non-endorsers  (*N* = 70) | | | | |  |
| --- | --- | --- | --- | --- | --- | --- | --- | --- | --- | --- | --- | --- |
|  | *n* | | % | | |  | *n* | | % | | |  |
| MI-Self Event | | 256 | | 73.1 | | |  | - | | - | | |
| MI-Other Event | | 280 | | 80.0 | | |  | - | | - | | |
| MI-Betrayal Event | | 295 | | 84.3 | | |  | - | | - | | |
| Worst Aspect | |  | |  | | |  |  | |  | | |
| MI-Self | | 160* | | 45.7 | | |  | - | | - | | |
| MI-Other | | 114 | | 32.6 | | |  | - | | - | | |
| MI-Betrayal | | 76** | | 21.7 | | |  | - | | - | | |
|  | | Criterion A Event  Endorsers  (*n* = 288) | | Criterion A Event Non-Endorsers  (*n*= 62) | | |  | Criterion A Event Endorsers  (*n* = 38) | | Criterion A Event Non-Endorsers  (*n* = 32) | | |
|  | | *n* | % | *n* | | % |  | *n* | % | *n* | | % |
| PTSD Screener  Symptoms | |  |  |  | |  |  |  |  |  | |  |
| Intrusions | | 246 | 85.4 | 31 | | 50.0 |  | 20 | 52.6 | 9 | | 28.1 |
| Avoidance | | 219 | 76.0 | 33 | | 53.2 |  | 18 | 47.4 | 8 | | 25.0 |
| Hyperarousal | | 200 | 69.4 | 37 | | 59.7 |  | 25 | 65.8 | 9 | | 28.1 |
| Numbing | | 180 | 62.5 | 30 | | 48.4 |  | 19 | 50.0 | 9 | | 28.1 |
| Guilt/Blame | | 178 | 61.8 | 31 | | 50.0 |  | 12 | 31.6 | 4 | | 12.5 |
| Number of PTSD  Symptoms Endorsed | |  |  |  | |  |  |  |  |  | |  |
| 0 | | 12 | 4.2 | 12 | | 19.4 |  | 7 | 18.4 | 19 | | 59.4 |
| 1 | | 6 | 2.1 | 5 | | 8.1 |  | 3 | 7.9 | 2 | | 6.3 |
| 2 | | 17 | 5.9 | 7 | | 11.3 |  | 5 | 13.2 | 4 | | 12.5 |
| 3 | | 103 | 35.8 | 18 | | 29.0 |  | 14 | 36.8 | 2 | | 6.3 |
| 4 | | 76 | 26.4 | 11 | | 17.7 |  | 6 | 15.8 | 2 | | 6.3 |
| 5 | | 74 | 25.7 | 9 | | 14.5 |  | 3 | 7.9 | 3 | | 9.4 |
|  | | Tests of Independence Between Criterion A Endorsers and Non-Endorsers | | | | | | | | | | |
|  | | *Χ^2^* | *p*-value | | % difference | |  | *Χ^2^* | *p*-value | | % difference | |
| PTSD Screener  Symptoms | |  |  | |  | |  |  |  | |  | |
| Intrusions | | 38.8 | <.001^+^ | | 35.4 | |  | 4.3 | .038^+^ | | 24.5 | |
| Avoidance | | 13.2 | <.001^+^ | | 22.8 | |  | 3.7 | .054 | | - | |
| Hyperarousal | | 2.2 | .136 | | - | |  | 9.9 | .002^+^ | | 37.7 | |
| Numbing | | 4.2 | .040^+^ | | 14.1 | |  | 3.5 | .063 | | - | |
| Guilt/Blame | | 3.0 | .086 | | - | |  | 3.6 | .058 | | - | |
| Number of PTSD  Symptoms Endorsed | |  |  | |  | |  |  |  | |  | |
| 0 | | 18.4 | <.001^+^ | | -15.2 | |  | 12.5 | <.001^+^ | | -41.0 | |
| 1 | | 6.0 | .014^+^ | | -6.0 | |  | 0.1 | .790 | | - | |
| 2 | | 2.3 | .128 | | - | |  | <0.1 | .935 | | - | |
| 3 | | 1.0 | .312 | | - | |  | 9.2 | .002^+^ | | 30.5 | |
| 4 | | 2.0 | .153 | | - | |  | 1.6 | .211 | | - | |
| 5 | | 3.5 | .060 | | - | |  | <0.1 | .826 | | - | |

**Table S12**

*Stage III Event Types and PTSD Screener Findings in US Sample* (*N* = 420)

*Note.* PMIE = Potentially Morally Injurious Event; *Difference in proportions for MI-Self and MI-Other: *χ2* = 12.002, *p* = .001, % difference = 12.8%; **Difference in proportions for MI-Other and MI-Betrayal: *χ2* = 11.685, *p* = .001, % difference = 11.5%; *Χ^2^*-test df = 1; ^+^*p*<.05.

|  | PMIE Endorsers  (*N* = 71) | | | | |  | PMIE Non-endorsers  (*N* = 40) | | | | |  |
| --- | --- | --- | --- | --- | --- | --- | --- | --- | --- | --- | --- | --- |
|  | *n* | | % | | |  | *n* | | % | | |  |
| MI-Self Event | | 45 | | 63.4 | | |  | - | | - | | |
| MI-Other Event | | 43 | | 60.6 | | |  | - | | - | | |
| MI-Betrayal Event | | 15 | | 21.1 | | |  | - | | - | | |
| Worst Aspect | |  | |  | | |  |  | |  | | |
| MI-Self | | 27 | | 38.0 | | |  | - | | - | | |
| MI-Other | | 28 | | 39.4 | | |  | - | | - | | |
| MI-Betrayal | | 16 | | 22.5 | | |  | - | | - | | |
|  | | Criterion A Event  Endorsers  (*n* = 27) | | Criterion A Event Non-Endorsers  (*n*= 44) | | |  | Criterion A Event Endorsers  (*n* = 15) | | Criterion A Event Non-Endorsers  (*n* = 25) | | |
|  | | *n* | % | *n* | | % |  | *n* | % | *n* | | % |
| PTSD Screener  Symptoms | |  |  |  | |  |  |  |  |  | |  |
| Intrusions | | 5 | 18.5 | 8 | | 18.2 |  | 1 | 6.7 | 0 | | 0.0 |
| Avoidance | | 9 | 33.3 | 3 | | 6.8 |  | 0 | 0.0 | 1 | | 4.0 |
| Hyperarousal | | 14 | 51.9 | 9 | | 20.5 |  | 3 | 20.0 | 5 | | 20.0 |
| Numbing | | 14 | 51.9 | 17 | | 38.6 |  | 4 | 26.7 | 5 | | 20.0 |
| Guilt/Blame | | 7 | 25.9 | 10 | | 22.7 |  | 0 | 0.0 | 2 | | 8.0 |
| Number of PTSD  Symptoms Endorsed | |  |  |  | |  |  |  |  |  | |  |
| 0 | | 5 | 18.5 | 24 | | 54.5 |  | 8 | 53.3 | 16 | | 64.0 |
| 1 | | 7 | 25.9 | 6 | | 13.6 |  | 6 | 40.0 | 5 | | 20.0 |
| 2 | | 6 | 22.2 | 5 | | 11.4 |  | 1 | 6.7 | 4 | | 16.0 |
| 3 | | 6 | 22.2 | 6 | | 13.6 |  | 0 | 0.0 | 0 | | 0.0 |
| 4 | | 3 | 11.1 | 2 | | 4.5 |  | 0 | 0.0 | 0 | | 0.0 |
| 5 | | 0 | 0.0 | 1 | | 2.3 |  | 0 | 0.0 | 0 | | 0.0 |
|  | | Tests of Independence Between Criterion A Endorsers and Non-Endorsers | | | | | | | | | | |
|  | | *Χ^2^* | *p*-value | | % difference | |  | *Χ^2^* | *p*-value | | % difference | |
| PTSD Screener  Symptoms | |  |  | |  | |  |  |  | |  | |
| Intrusions | | <0.1 | .972 | | - | |  | 1.7 | .191 | | - | |
| Avoidance | | 8.4 | .004^+^ | | 26.5 | |  | 0.6 | .433 | | - | |
| Hyperarousal | | 7.5 | .006^+^ | | 5.0 | |  | 0.0 | - | | - | |
| Numbing | | 1.2 | .276 | | - | |  | 0.2 | .625 | | - | |
| Guilt/Blame | | 0.1 | .759 | | - | |  | 1.3 | .261 | | - | |
| Number of PTSD  Symptoms Endorsed | |  |  | |  | |  |  |  | |  | |
| 0 | | 9.0 | .003^+^ | | -36.0 | |  | .44 | .505 | | - | |
| 1 | | 1.7 | .194 | | - | |  | 1.9 | .170 | | - | |
| 2 | | 1.5 | .220 | | - | |  | 0.7 | .388 | | - | |
| 3 | | 0.9 | .349 | | - | |  | - | - | | - | |
| 4 | | 1.1 | .294 | | - | |  | - | - | | - | |
| 5 | | 0.6 | .430 | | - | |  | - | - | | - | |

**Table S13**

*Stage III Event Types and PTSD Screener Findings in Israeli Sample* (*N* = 111)

*Note.* PMIE = Potentially Morally Injurious Event; *Difference in proportions for MI-Self and MI-Other: *χ2* <.001, *p* = 1.00, % difference = 1.4%; **Difference in proportions for MI-Other and MI-Betrayal: *χ2* = 3.985, *p* = .05, % difference = 16.9%; *Χ^2^*-test df = 1; ^+^*p*<.05.

|  | PMIE Endorsers  (*N* = 91) | | | | |  | PMIE Non-endorsers  (*N* = 13) | | | | |  |
| --- | --- | --- | --- | --- | --- | --- | --- | --- | --- | --- | --- | --- |
|  | *n* | | % | | |  | *n* | | % | | |  |
| MI-Self Event | | 40 | | 44.0 | | |  | - | | - | | |
| MI-Other Event | | 67 | | 73.6 | | |  | - | | - | | |
| MI-Betrayal Event | | 71 | | 78.0 | | |  | - | | - | | |
| Worst Aspect | |  | |  | | |  |  | |  | | |
| MI-Self | | 22 | | 24.2 | | |  | - | | - | | |
| MI-Other | | 47 | | 51.6 | | |  | - | | - | | |
| MI-Betrayal | | 21 | | 23.1 | | |  | - | | - | | |
|  | | Criterion A Event  Endorsers  (*n* = 55) | | Criterion A Event Non-Endorsers  (*n*= 34) | | |  | Criterion A Event Endorsers  (*n* = 8) | | Criterion A Event Non-Endorsers  (*n* = 5) | | |
|  | | *n* | % | *n* | | % |  | *n* | % | *n* | | % |
| PTSD Screener  Symptoms | |  |  |  | |  |  |  |  |  | |  |
| Intrusions | | 31 | 56.4 | 14 | | 41.2 |  | - | - | - | | - |
| Avoidance | | 43 | 78.2 | 21 | | 61.8 |  | - | - | - | | - |
| Hyperarousal | | 34 | 61.8 | 18 | | 52.9 |  | - | - | - | | - |
| Numbing | | 41 | 74.5 | 19 | | 55.9 |  | - | - | - | | - |
| Guilt/Blame | | 35 | 63.6 | 17 | | 50.0 |  | - | - | - | | - |
| Number of PTSD  Symptoms Endorsed | |  |  |  | |  |  |  |  |  | |  |
| 0 | | 6 | 10.9 | 8 | | 23.5 |  | - | - | - | | - |
| 1 | | 2 | 3.6 | 5 | | 14.7 |  | - | - | - | | - |
| 2 | | 8 | 14.5 | 2 | | 5.9 |  | - | - | - | | - |
| 3 | | 8 | 14.5 | 5 | | 14.7 |  | - | - | - | | - |
| 4 | | 13 | 23.6 | 5 | | 14.7 |  | - | - | - | | - |
| 5 | | 18 | 32.7 | 9 | | 26.5 |  | - | - | - | | - |
|  | | Tests of Independence Between Criterion A Endorsers and Non-Endorsers | | | | | | | | | | |
|  | | *Χ^2^* | *p*-value | | % difference | |  | *Χ^2^* | *p*-value | | % difference | |
| PTSD Screener  Symptoms | |  |  | |  | |  |  |  | |  | |
| Intrusions | | 1.9 | .164 | |  | |  | - | - | | - | |
| Avoidance | | 2.8 | .094 | |  | |  | - | - | | - | |
| Hyperarousal | | 0.7 | .409 | |  | |  | - | - | | - | |
| Numbing | | 3.3 | .068 | |  | |  | - | - | | - | |
| Guilt/Blame | | 1.6 | .205 | |  | |  | - | - | | - | |
| Number of PTSD  Symptoms Endorsed | |  |  | |  | |  |  |  | |  | |
| 0 | | 2.5 | .112 | |  | |  | - | - | | - | |
| 1 | | 3.6 | .059 | |  | |  | - | - | | - | |
| 2 | | 1.6 | .209 | |  | |  | - | - | | - | |
| 3 | | <0.1 | .983 | |  | |  | - | - | | - | |
| 4 | | 1.0 | .308 | |  | |  | - | - | | - | |
| 5 | | 0.4 | .533 | |  | |  | - | - | | - | |

**Table S14**

*Stage III Event Types and PTSD Screener Findings in Australian Sample* (*N* = 145)

*Note.* PMIE = Potentially Morally Injurious Event; *Difference in proportions for MI-Self and MI-Other: *χ2* = 13.445, *p* <.001, % difference = 27.4%; **Difference in proportions for MI-Other and MI-Betrayal: *χ2* = 14.674, *p* <.001, % difference = 28.5%; *Χ^2^*-test df = 1; ^+^*p*<.05.

**Table S15**

*Descriptive Statistics and Correlations for Study Variables Among PMIE-Endorsers in the US Stage III Sample.*

| Variable | *N* | *M* | *SD* | 1 | 2 | 3 | 4 | 5 | 6 | 7 | 8 | 9 | 10 | 11 | 12 | 13 | 14 | 15 | 16 |
| --- | --- | --- | --- | --- | --- | --- | --- | --- | --- | --- | --- | --- | --- | --- | --- | --- | --- | --- | --- |
| 1. MIOS Total Score | 350 | 33.59 | 13.37 | - |  |  |  |  |  |  |  |  |  |  |  |  |  |  |  |
| 2. MIOS Shame Subscale | 350 | 16.51 | 7.28 | .966** | - |  |  |  |  |  |  |  |  |  |  |  |  |  |  |
| 3. MIOS Trust Violation Subscale | 350 | 17.08 | 6.62 | .958** | .851** | - |  |  |  |  |  |  |  |  |  |  |  |  |  |
| 4. PCL-5 | 349 | 50.05 | 19.41 | .729** | .705** | .698** | - |  |  |  |  |  |  |  |  |  |  |  |  |
| 5. PHQ-9 | 350 | 15.35 | 6.59 | .619** | .619** | .569** | .719** | - |  |  |  |  |  |  |  |  |  |  |  |
| 6. B-IPF | 350 | 30.50 | 9.06 | .717** | .690** | .689** | .726** | .686** | - |  |  |  |  |  |  |  |  |  |  |
| 7. TRGI | 350 | 2.09 | .68 | .301** | .363** | .209** | .424** | .296** | .306** | - |  |  |  |  |  |  |  |  |  |
| 8. SSGS | 350 | 34.77 | 10.24 | .696** | .732** | .602** | .734** | .709** | .716** | .535** | - |  |  |  |  |  |  |  |  |
| 9. RSS Scale | 350 | 3.42 | .98 | .685** | .707** | .607** | .807** | .731** | .699** | .459** | .819** | - |  |  |  |  |  |  |  |
| 10. DAR-5 | 350 | 16.28 | 5.41 | .711** | .684** | .685** | .748** | .688** | .725** | .370** | .730** | .751** | - |  |  |  |  |  |  |
| 11. EMIS-M Self Subscale | 350 | 30.91 | 8.43 | .744** | .745** | .684** | .811** | .639** | .672** | .461** | .741** | .760** | .710** | - |  |  |  |  |  |
| 12. EMIS-M Other Subscale | 350 | 29.13 | 7.15 | .643** | .587** | .654** | .669** | .587** | .625** | .235** | .562** | .585** | .605** | .799** | - |  |  |  |  |

*Note*. B-IPF = Brief Inventory of Psychosocial Functioning. DAR = Dimensions of Anger Reactions. EMIS-M = Expressions of Moral Injury Scale – Military Version. MIOS = Moral Injury Outcomes Scale. PCL-5 = PTSD Checklist for DSM-5. PMIE = Potentially Morally Injurious Event. PHQ-9 = Patient Health Questionnaire-9. RSS = Religious and Spiritual Struggles. SSGS = State Shame and Guilt Scale. TRGI = Trauma-Related Guilt Inventory. * *p* < .05; ** *p* < .01

**Table S16**

*Descriptive Statistics and Correlations for Study Variables Among PMIE-Non-Endorsers in the Stage III US Sample*

| Variable | *N* | *M* | *SD* | 1 | 2 | 3 | 4 | 5 | 6 | 7 | 8 | 9 | 10 | 11 | 12 | 13 | 14 | 15 |
| --- | --- | --- | --- | --- | --- | --- | --- | --- | --- | --- | --- | --- | --- | --- | --- | --- | --- | --- |
| 1. MIOS Total Score | 70 | 20.90 | 13.27 | - |  |  |  |  |  |  |  |  |  |  |  |  |  |  |
| 2. MIOS Shame Subscale | 70 | 9.93 | 6.87 | .972^**^ | - |  |  |  |  |  |  |  |  |  |  |  |  |  |
| 3. MIOS Trust Violation Subscale | 70 | 10.97 | 6.80 | .971^**^ | .887^**^ | - |  |  |  |  |  |  |  |  |  |  |  |  |
| 4. PCL-5 | 70 | 28.83 | 21.84 | .669^**^ | .675^**^ | .624^**^ | - |  |  |  |  |  |  |  |  |  |  |  |
| 5. PHQ-9 | 70 | 8.06 | 6.40 | .667^**^ | .661^**^ | .635^**^ | .765^**^ | - |  |  |  |  |  |  |  |  |  |  |
| 6. B-IPF | 65 | 48.89 | 33.06 | .474^*^ | .457^**^ | .461^**^ | .631^**^ | .573^**^ | - |  |  |  |  |  |  |  |  |  |
| 7. TRGI | 70 | 1.35 | .69 | .413^*^ | .465^**^ | .336^**^ | .487^**^ | .509^**^ | .374^**^ | - |  |  |  |  |  |  |  |  |
| 8. SSGS | 70 | 24.00 | 12.48 | .740^**^ | .726^**^ | .712^**^ | .673^**^ | .702^**^ | .570^**^ | .478^**^ | - |  |  |  |  |  |  |  |
| 9. RSS Scale | 70 | 2.22 | 1.12 | .623^**^ | .625^**^ | .585^**^ | .726^**^ | .690^**^ | .693^**^ | .636^**^ | .762^**^ | - |  |  |  |  |  |  |
| 10. DAR-5 | 70 | 10.97 | 4.80 | .680^**^ | .654^**^ | .666^**^ | .676^**^ | .785^**^ | .541^**^ | .552^**^ | .721^**^ | .671^**^ | - |  |  |  |  |  |
| 12. EMIS-M Self Subscale | 70 | 22.10 | 9.54 | .794^**^ | .785^**^ | .756^**^ | .745^**^ | .745^**^ | .624^**^ | .507^**^ | .719^**^ | .743^**^ | .776^**^ | - |  |  |  |  |
| 13. EMIS-M Other Subscale | 70 | 20.83 | 8.33 | .733^**^ | .730^**^ | .695^**^ | .643^**^ | .644^**^ | .589^**^ | .354^**^ | .653^**^ | .680^**^ | .670^**^ | .897^**^ | - |  |  |  |

*Note*. B-IPF = Brief Inventory of Psychosocial Functioning. DAR-5 = Dimensions of Anger Reactions-5. EMIS-M = Expressions of Moral Injury Scale – Military Version. MIOS = Moral Injury Outcomes Scale. PCL-5 = PTSD Checklist for DSM-5. PMIE = Potentially Morally Injurious Event. PHQ-9 = Patient Health Questionnaire-9. RSS = Religious and Spiritual Struggles. SSGS = State Shame and Guilt Scale. TRGI = Trauma-Related Guilt Inventory. * *p* < .05; ** *p* < .01

**Table S17**

*Descriptive Statistics and Correlations for Study Variables Among PMIE-Endorsers in the Israeli Sample.*

| Variable | *N* | *M* | *SD* | 1 | 2 | 3 | 4 | 5 | 6 | 7 | 8 | 9 | 10 | 11 | 12 | 13 | 14 | 15 | 16 |
| --- | --- | --- | --- | --- | --- | --- | --- | --- | --- | --- | --- | --- | --- | --- | --- | --- | --- | --- | --- |
| 1. MIOS Total Score | 71 | 14.55 | 9.28 | - |  |  |  |  |  |  |  |  |  |  |  |  |  |  |  |
| 2. MIOS Shame Subscale | 71 | 5.96 | 5.20 | .886^**^ | - |  |  |  |  |  |  |  |  |  |  |  |  |  |  |
| 3. MIOS Trust Violation Subscale | 71 | 8.59 | 5.25 | .889^**^ | .575^**^ | - |  |  |  |  |  |  |  |  |  |  |  |  |  |
| 4. PCL-5 | 71 | 20.01 | 16.48 | .574^**^ | .587^**^ | .433^**^ | - |  |  |  |  |  |  |  |  |  |  |  |  |
| 5. PHQ-9 | 71 | 2.51 | 3.84 | .486^**^ | .475^**^ | .388^**^ | .488^**^ | - |  |  |  |  |  |  |  |  |  |  |  |
| 6. B-IPF | 69 | 22.96 | 24.84 | .441^**^ | .327^**^ | .455^**^ | .618^**^ | .299^*^ | - |  |  |  |  |  |  |  |  |  |  |
| 7. TRGI | 71 | 1.36 | .78 | .403^**^ | .536^**^ | .181 | .266^*^ | .126 | .091 | - |  |  |  |  |  |  |  |  |  |
| 8. SSGS | 71 | 19.44 | 8.75 | .687^**^ | .673^**^ | .546^**^ | .676^**^ | .708^**^ | .460^**^ | .365^**^ | - |  |  |  |  |  |  |  |  |
| 9. RSS Scale | 71 | 2.20 | 1.01 | .667^**^ | .574^**^ | .610^**^ | .522^**^ | .574^**^ | .356^**^ | .353^**^ | .751^**^ | - |  |  |  |  |  |  |  |
| 10. DAR-5 | 71 | 11.20 | 4.62 | .664^**^ | .573^**^ | .606^**^ | .585^**^ | .601^**^ | .377^**^ | .225 | .638^**^ | .662^**^ | - |  |  |  |  |  |  |
| 11. EMIS-M Self Subscale | 71 | 15.30 | 5.97 | .782^**^ | .755^**^ | .633^**^ | .582^**^ | .367^**^ | .493^**^ | .480^**^ | .601^**^ | .607^**^ | .479^**^ | - |  |  |  |  |  |
| 12. EMIS-M Other Subscale | 71 | 19.75 | 8.63 | .718^**^ | .587^**^ | .687^**^ | .519^**^ | .340^**^ | .457^**^ | .324^**^ | .532^**^ | .612^**^ | .584^**^ | .704^**^ | - |  |  |  |  |

*Note*. B-IPF = Brief Inventory of Psychosocial Functioning. DAR-5 = Dimensions of Anger Reactions-5. EMIS-M = Expressions of Moral Injury Scale – Military Version. MIOS = Moral Injury Outcomes Scale. PCL-5 = PTSD Checklist for DSM-5. PMIE = Potentially Morally Injurious Event. PHQ-9 = Patient Health Questionnaire-9. RSS = Religious and Spiritual Struggles. SSGS = State Shame and Guilt Scale. TRGI = Trauma-Related Guilt Inventory. * *p* < .05; ** *p* < .01

**Table S18**

| Variable | *N* | *M* | *SD* | 1 | 2 | 3 | 4 | 5 | 6 | 7 | 8 | 9 | 10 | 11 | 12 | 13 | 14 | 15 |
| --- | --- | --- | --- | --- | --- | --- | --- | --- | --- | --- | --- | --- | --- | --- | --- | --- | --- | --- |
| 1. MIOS Total Score | 40 | 5.95 | 6.80 | - |  |  |  |  |  |  |  |  |  |  |  |  |  |  |
| 2. MIOS Shame Subscale | 40 | 2.45 | 3.78 | .900^**^ | - |  |  |  |  |  |  |  |  |  |  |  |  |  |
| 3. MIOS Trust Violation Subscale | 40 | 3.50 | 3.78 | .900^**^ | .619^**^ | - |  |  |  |  |  |  |  |  |  |  |  |  |
| 4. PCL-5 | 40 | 5.43 | 7.11 | .422^**^ | .339^*^ | .421^**^ | - |  |  |  |  |  |  |  |  |  |  |  |
| 5. PHQ-9 | 40 | .70 | 1.60 | .304 | .243 | .304 | .243 | - |  |  |  |  |  |  |  |  |  |  |
| 6. B-IPF | 37 | 8.07 | 19.25 | .375* | .291 | .381^*^ | .515^**^ | -.023 | - |  |  |  |  |  |  |  |  |  |
| 7. TRGI | 40 | .92 | .68 | .347* | .542^**^ | .083 | .124 | -.084 | .091 | - |  |  |  |  |  |  |  |  |
| 8. SSGS | 40 | 12.78 | 5.07 | .672^**^ | .567^**^ | .642^**^ | .671^**^ | .430^**^ | .665^**^ | .204 | - |  |  |  |  |  |  |  |
| 9. RSS Scale | 40 | 1.70 | .78 | .453^**^ | .228 | .586^**^ | .379^*^ | .129 | .517^**^ | .138 | .585^**^ | - |  |  |  |  |  |  |
| 10. DAR-5 | 40 | 7.87 | 3.54 | .545^**^ | .419^**^ | .562^**^ | .517^**^ | .580^**^ | .375^*^ | .040 | .631^**^ | .461^**^ | - |  |  |  |  |  |
| 12. EMIS-M Self Subscale | 40 | 10.40 | 3.59 | .464^**^ | .465^**^ | .370^*^ | .683^**^ | -.094 | .721^**^ | .303 | .601^**^ | .349^*^ | .277 | - |  |  |  |  |
| 13. EMIS-M Other Subscale | 40 | 12.25 | 6.57 | .578^**^ | .414^**^ | .626^**^ | .446^**^ | .358^*^ | .292 | -.035 | .569^**^ | .380^**^ | .452^**^ | .428^**^ | - |  |  |  |

*Descriptive Statistics and Correlations for Study Variables Among PMIE-Non-Endorsers in the Israeli Sample.*

*Note*. B-IPF = Brief Inventory of Psychosocial Functioning. DAR-5 = Dimensions of Anger Reactions-5. EMIS-M = Expressions of Moral Injury Scale – Military Version. MIOS = Moral Injury Outcomes Scale. PCL-5 = PTSD Checklist for DSM-5. PMIE = Potentially Morally Injurious Event. PHQ-9 = Patient Health Questionnaire-9. RSS = Religious and Spiritual Struggles. SSGS = State Shame and Guilt Scale. TRGI = Trauma-Related Guilt Inventory. * *p* < .05; ^**^ *p* < .01

**Table S19**

*Descriptive Statistics and Correlations for Study Variables among PMIE-Endorsers - Australia*

| Variable | *N* | *M* | *SD* | 1 | 2 | 3 | 4 | 5 | 6 | 7 | 8 | 9 | 10 | 11 | 12 | 13 | 14 | 15 |
| --- | --- | --- | --- | --- | --- | --- | --- | --- | --- | --- | --- | --- | --- | --- | --- | --- | --- | --- |
| 1. MIOS Total Score | 87 | 27.30 | 9.98 | - |  |  |  |  |  |  |  |  |  |  |  |  |  |  |
| 2. MIOS Shame Subscale | 87 | 11.80 | 6.07 | .864^**^ | - |  |  |  |  |  |  |  |  |  |  |  |  |  |
| 3. MIOS Trust Violation Subscale | 87 | 15.49 | 5.64 | .840^**^ | .451^**^ | - |  |  |  |  |  |  |  |  |  |  |  |  |
| 4. PCL-5 | 84 | 58.95 | 19.22 | .631^**^ | .496^**^ | .567^**^ | - |  |  |  |  |  |  |  |  |  |  |  |
| 5. PHQ-9 | 79 | 11.76 | 6.74 | .520^**^ | .406^**^ | .458^**^ | .774^**^ | - |  |  |  |  |  |  |  |  |  |  |
| 6. B-IPF | 87 | 53.40 | 26.69 | .606^**^ | .583^**^ | .441^**^ | .671^**^ | .584^**^ | - |  |  |  |  |  |  |  |  |  |
| 7. TRGI | 77 | 1.39 | 0.77 | .379^**^ | .580^**^ | .026 | .206 | .064 | .222 | - |  |  |  |  |  |  |  |  |
| 8. SSGS | 84 | 22.54 | 9.22 | .541^**^ | .660^**^ | .228^*^ | .590^**^ | .540^**^ | .457^**^ | .502^**^ | - |  |  |  |  |  |  |  |
| 9. RSS Scale | 72 | 18.54 | 8.26 | .513^**^ | .457^**^ | .386^**^ | .545^**^ | .577^**^ | .369^**^ | .453^**^ | .539^**^ | - |  |  |  |  |  |  |
| 10. DAR | 82 | 12.40 | 6.00 | .634^**^ | .538^**^ | .525^**^ | .687^**^ | .472^**^ | .568^**^ | .160 | .521^**^ | .358^**^ | - |  |  |  |  |  |
| 11. EMIS-M Self Subscale | 70 | 23.17 | 7.43 | .686^**^ | .588^**^ | .547^**^ | .685^**^ | .626^**^ | .661^**^ | .364^**^ | .628^**^ | .675^**^ | .528^**^ | - |  |  |  |  |
| 12. EMIS-M Other Subscale | 70 | 28.19 | 7.44 | .479^**^ | .249^**^ | .558^**^ | .513^**^ | .410^**^ | .522^**^ | -.016 | .217 | .357^**^ | .436^**^ | .581^**^ | - |  |  |  |

*Note*. B-IPF = Brief Inventory of Psychosocial Functioning. DAR = Dimensions of Anger Reactions. EMIS = Expressions of Moral Injury Scale – Military Version. MIOS = Moral Injury Outcomes Scale. PCL-5 = PTSD Checklist for DSM-5. PMIE = Potentially Morally Injurious Event. PHQ-9 = Patient Health Questionnaire-9. RSS = Religious and Spiritual Struggles Scale. SSGS = State Shame and Guilt Scale. TRGI = Trauma-Related Guilt Inventory. * p < .05; ** p < .01

*
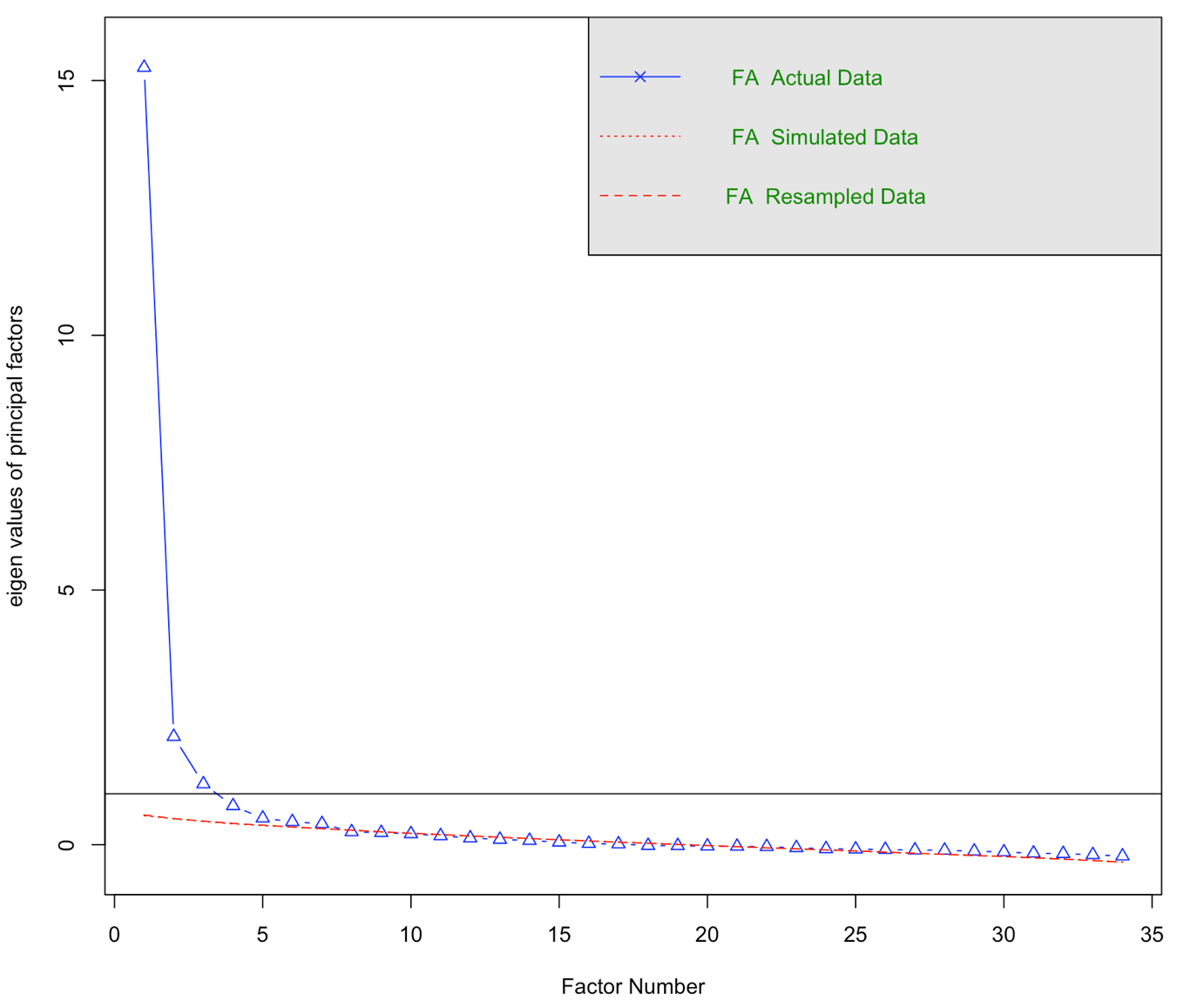
*

*Figure 1.* Parallel analysis of preliminary 34-item MIOS.
